# DNA methylation aging and transcriptomic studies in horses

**DOI:** 10.1038/s41467-021-27754-y

**Published:** 2022-01-10

**Authors:** Steve Horvath, Amin Haghani, Sichong Peng, Erin N. Hales, Joseph A. Zoller, Ken Raj, Brenda Larison, Todd R. Robeck, Jessica L. Petersen, Rebecca R. Bellone, Carrie J. Finno

**Affiliations:** 1 Department of Human Genetics, David Geffen School of Medicine, University of California, Los Angeles, Los Angeles, CA USA; 2 Department of Biostatistics, Fielding School of Public Health, University of California, Los Angeles, Los Angeles, CA USA; 3 Department of Population Health and Reproduction, University of California, Davis School of Veterinary Medicine, Davis, CA USA; 4 Radiation Effects Department, Centre for Radiation, Chemical and Environmental Hazards, Public Health England, Chilton, Didcot UK; 5 Department of Ecology and Evolutionary Biology, University of California, Los Angeles, CA USA; 6 Center for Tropical Research, Institute of the Environment and Sustainability, University of California, Los Angeles, CA USA; 7 Zoological Operations, SeaWorld Parks and Entertainment, 7007 SeaWorld Drive, Orlando, FL USA; 8 Department of Animal Science, University of Nebraska, Lincoln, NE USA; 9 Veterinary Genetics Laboratory, University of California, Davis School of Veterinary Medicine, Davis, CA USA

**Keywords:** Predictive markers, Ageing, DNA methylation

## Abstract

Cytosine methylation patterns have not yet been thoroughly studied in horses. Here, we profile *n* = 333 samples from 42 horse tissue types at loci that are highly conserved between mammalian species using a custom array (HorvathMammalMethylChip40). Using the blood and liver tissues from horses, we develop five epigenetic aging clocks: a multi-tissue clock, a blood clock, a liver clock and two dual-species clocks that apply to both horses and humans. In addition, using blood methylation data from three additional equid species (plains zebra, Grevy’s zebras and Somali asses), we develop another clock that applies across all equid species. Castration does not significantly impact the epigenetic aging rate of blood or liver samples from horses. Methylation and RNA data from the same tissues define the relationship between methylation and RNA expression across horse tissues. We expect that the multi-tissue atlas will become a valuable resource.

## Abstract (web summary)

Methylation levels of specific sites in the genome is correlated with aging. Here the authors develop a human-horse clock which could assist in translating anti-aging interventions from humans to horses and vice versa.

## Introduction

It has long been known that the level of cellular DNA methylation changes with age^1–3^. With the technical development of methylation arrays that profile large numbers of individual CpG positions in the genome, an opportunity arose to develop a highly accurate age-estimator for all human tissues^4–6^. For example, the human pan-tissue clock combines the weighted average of methylation levels of 353 CpGs into an age estimate referred to as DNAm age or epigenetic age^7^. While the human pan-tissue clock applies to chimpanzees,^7^ it does not apply to more distantly related mammals as a result of evolutionary genome sequence divergence. Epigenetic clocks have been developed for mice and many other species^8–13^. Overall, these publications indicate that the underlying biological principle of epigenetic clocks is shared between members of different mammalian species. In humans, the discrepancy between DNA methylation age and chronological age (which is termed “epigenetic age acceleration”) is predictive of multiple health conditions^14–19^. Epigenetic age is predictive of mortality, even after adjusting for known risk factors such as chronological age, sex, smoking status, and other risk factors^14–19^. Collectively, the evidence is compelling that epigenetic age is an indicator of biological age^4–6,20–23^. Human epigenetic clocks have already found many biomedical applications, including the measure of biological age in human anti-aging clinical trials^4,24^.

Here, we develop epigenetic clocks for horses and other equid species. Our human–horse clock was developed for a futuristic goal: to translate anti-aging interventions from humans to horses and vice versa. We characterize changes in DNA methylation that accompany equine aging and castration. To study the relationship between expression levels (mRNA) and methylation across tissue types, we generated DNA methylation profiles from across 42 horse tissues for which RNA-seq profiles were also available.

## Results

We generated DNA methylation profiles from various tissue samples from domestic horses (Table 1). The horse methylation data were used for two broad categories of analyses: (1) epigenetic aging studies in blood and liver, (2) comparing DNAm levels to transcriptomic data across 42 different tissues types. The aging studies in horses used *N* = 192 blood samples and *N* = 48 liver samples from multiple horse breeds aged between 0 and 29 years. By contrast, the multi-tissue atlas involved 42 different tissues from *N* = 2 mares used in the equine Functional Annotation of Animal Genomes (FAANG) initiative^25^.

**Table 1:** Description of blood methylation data.

Tissue	*N*	No. of female	No. of male castrated	Breed	Mean age	Min. age	Max. age
*Clocks*							
Blood	192	131	49	Multiple	11.2	0.005	28
Liver	48	24	15	Multiple	4.42	0.042	29
*Tissue atlas*							
Adipose	2	2	NA	TB	4.5	4	5
AdrenalCortex	2	2	NA	TB	4.5	4	5
Cartilage	2	2	NA	TB	4.5	4	5
Cecum	2	2	NA	TB	4.5	4	5
Cerebellum^a^	4	4	NA	TB	4.5	4	5
CerebralCortex^b^	6	6	NA	TB	4.5	4	5
Duodenum	2	2	NA	TB	4.5	4	5
Fibroblast	2	2	NA	TB	4.5	4	5
Heart^c^	8	8	NA	TB	4.5	4	5
Hypothalamus	2	2	NA	TB	4.5	4	5
Ileum	2	2	NA	TB	4.5	4	5
Jejunum	2	2	NA	TB	4.5	4	5
Keratinocyte	2	2	NA	TB	4.5	4	5
Kidney^d^	4	4	NA	TB	4.5	4	5
Lamina	2	2	NA	TB	4.5	4	5
Larynx	2	2	NA	TB	4.5	4	5
Lung	2	2	NA	TB	4.5	4	5
Mammary	2	2	NA	TB	4.5	4	5
MitralValve	2	2	NA	TB	4.5	4	5
Muscle^e^	8	8	NA	TB	4.5	4	5
Ovary	2	2	NA	TB	4.5	4	5
Pituitary	2	2	NA	TB	4.5	4	5
Skin	2	2	NA	TB	4.5	4	5
SpinalCord^f^	4	4	NA	TB	4.5	4	5
Spleen	2	2	NA	TB	4.5	4	5
SuspensoryLig	2	2	NA	TB	4.5	4	5
Tendon^g^	4	4	NA	TB	4.5	4	5
Uterus	2	2	NA	TB	4.5	4	5

Total # Unique tissues = 42.

*N* = total number of samples per species. Number of females. Age: mean, minimum and maximum. *NA* = not applicable.

^a^Lateral hemisphere and vermis.

^b^Frontal cortex, parietal cortex and temporal cortex.

^c^Left atrium, left ventricle, right atrium and right ventricle.

^d^Kidney cortex and kidney medulla.

^e^Gluteal muscle, two regions of longissimus muscle, sacrocaudalis dorsalis muscle.

^f^C1 and T8 spinal cord.

^g^Superficial digital flexor tendon and deep digital flexor tendon.

Unsupervised hierarchical clustering analysis of these profiles led to distinct tissue-based clusters (color band in Fig. S1). A subsequent random forest analysis of sex led to an error rate of 0% according to the out-of-bag (OOB) estimates.

### Epigenetic clocks

**Fig. 1:**
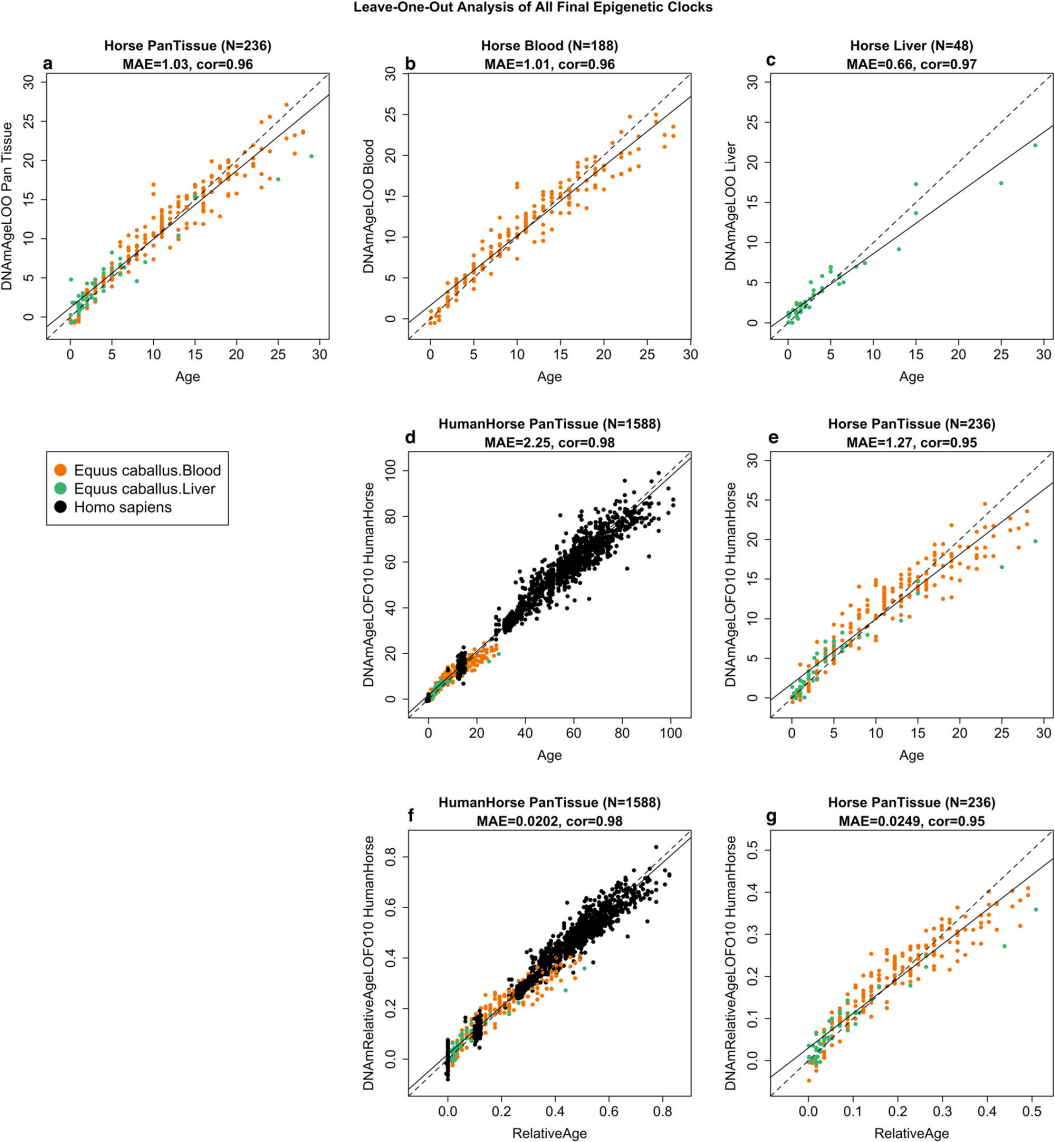
Cross-validation study of epigenetic clocks for horses and humans. Chronological age (*x*-axis) versus leave-one-sample-out (LOSO) estimate of DNA methylation age (*y*-axis, in units of years) for **a** the multi-tissue clock for horse blood and liver, **b** horse blood clock, **c** horse liver clock. **d** Ten-fold cross-validation (LOFO10) analysis of the human-horse clock for chronological age. Dots are colored by species (black = human) and horse tissue type (green = liver, orange = blood). **e** Same as panel **d** but restricted to horses. **f** Ten-fold cross-validation analysis of the human–horse clock for relative age, which is the ratio of chronological age to the maximum recorded lifespan of the respective species. **g** The same as panel **d** but restricted to horses. Each panel reports sample size, correlation coefficient, median absolute error (MAE).

Using the blood and liver tissues from horses, we developed six epigenetic clocks: a multi-tissue clock, a blood clock, a liver clock, and two dual-species clocks that apply to both horses and humans. Using blood methylation data from three additional equid species (plains zebra, Grevy’s zebras, and Somali asses), we developed another clock that applies to all equid species. To develop the pure horse clocks, the training data employed consisted of horse blood and/or liver DNA methylation profiles, while human and horse DNA methylation profiles constituted the training data for both the human–horse clocks. To arrive at unbiased estimates of the epigenetic clocks, we carried out a cross-validation analysis of the training data. The cross-validation study reports unbiased estimates of the age correlation R (defined as Pearson correlation between chronological age and its estimate, DNAm age), as well as the median absolute error. As indicated by its name, the horse multi-tissue clock is highly accurate in age estimation of blood and liver (*R* = 0.96 and median absolute error 1.0 years, Fig. 1a). The horse clocks for blood and liver samples lead to similarly high levels of accuracy (Fig. 1b, c).

The dual-species (human–horse) epigenetic clocks were trained using horse and human DNA methylation data. The resulting two human–horse clocks mutually differ by way of age measurement. One estimates the ages of horses and humans (in units of years, Fig. 1d, e), while the other estimates *relative* ages (Fig. 1f, g). Relative age is the ratio of the chronological age of an animal to the maximum lifespan of its species (122.5 for humans and 57 years for horses, Supplementary Table 1, see the “Methods” section). The relative age ratio (with resulting values between 0 and 1) allows alignment and biologically meaningful comparison between species with different lifespans, which cannot otherwise be afforded by direct comparison of their chronological ages.

The human–horse clock for chronological age is highly accurate when DNA methylation profiles of both species are analyzed together (*R* = 0.98, Fig. 1d), and remains remarkably accurate when restricted to horse blood and liver samples (*R* = 0.95, Fig. 1e). Similarly, the human-horse clock for relative *age* exhibits a high correlation regardless of whether the analysis is applied to samples from both species (*R* = 0.98, Fig. 1f) or only to horse samples (*R* = 0.94, Fig. 1g). This demonstrates that relative age circumvents the skewing that is inherent when the chronological age of species with different lifespans is measured using a single formula.

### Equid clock

**Fig. 2:**
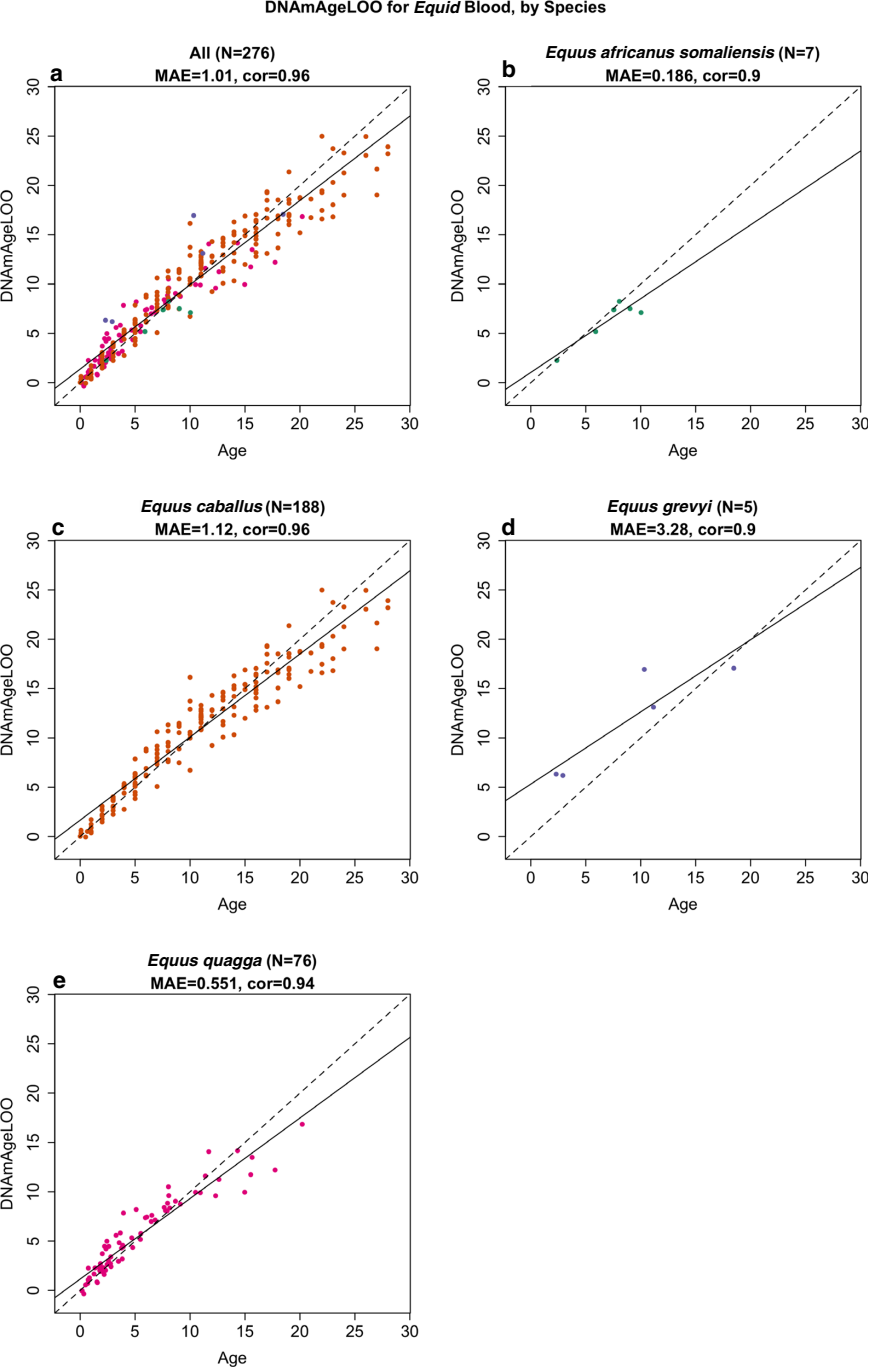
The equid clock for blood samples. Blood samples (dots) are colored by species as indicated in the respective panels. Leave one sample out cross-validation estimate of age (*y*-axis) versus chronological age in **a** all species combined, **b** *Equus africanus somaliensis*, **c** *Equus caballus*, **d** *Equus grevyi*, **e** *Equus quagga*. Each panel reports the number of blood samples, median absolute error in units of years, and Pearson correlation.

Before building an equid clock, we first applied the horse clocks to plains zebras (*Equus quagga*) since we had the largest numbers of samples from this species (*N* = 76 blood samples and N20 biopsy skin samples). According to the five different horse clocks, the DNAmAge estimates from plains zebra blood correlate highly with the age of the zebra (Fig. S2). However, these clocks performed poorly in skin biopsy samples leading to large median errors (Fig. S3). Based on these results, we decided to build our equid clock on the basis of blood samples only. The equid clock was trained on blood samples from four equids: domestic horse (*N* = 188), plains zebras (*N* = 76), Grevy’s zebra *N* = 5, and Somali wild ass (*N* = 7). To evaluate the accuracy of the equid clock, we carried out two cross-validation schemes that serve different purposes. First, the leave-one-sample-out (LOO) cross-validation analysis estimates accuracy in blood samples from the four species. We find that the equid blood clock is highly accurate across four equid species (LOO estimate *R* = 0.96, median error 1.0 years, Fig. 2a). The equid clock performs well in each of the underlying species (*R* = 0.90 in *Equus africanus somaliensis*, Fig. 2b), *Equus caballus* (*R* = 0.96, Fig. 2c), *Equus grevyi* (*R* = 0.90, Fig. 2d), *Equus quagga* (*R* = 0.94, Fig. 2e).

The second cross-validation scheme, leave-one-species out (LOSO) analysis, attempts to estimate the accuracy in (future) equid species that were not part of the original training set. Again, we find a high age correlation (LOSO estimate *R* ≥ 0.91 across the four equid species, median error = 1.5 years, Fig. S4). However, the DNAm age estimate is expected to over or underestimate the true chronological age in novel equid species, as exemplified by the LOSO estimate in Equus grevyi, where the over-estimate results in a high median error = 4.6 years despite the strong age correlation (*r* = 0.93, Fig. S4D). The systematic bias/offset between DNAmAge and age can be estimated by adding blood samples from animals of known age to the test data set.

### EWAS of age in horse tissues

**Fig. 3:**
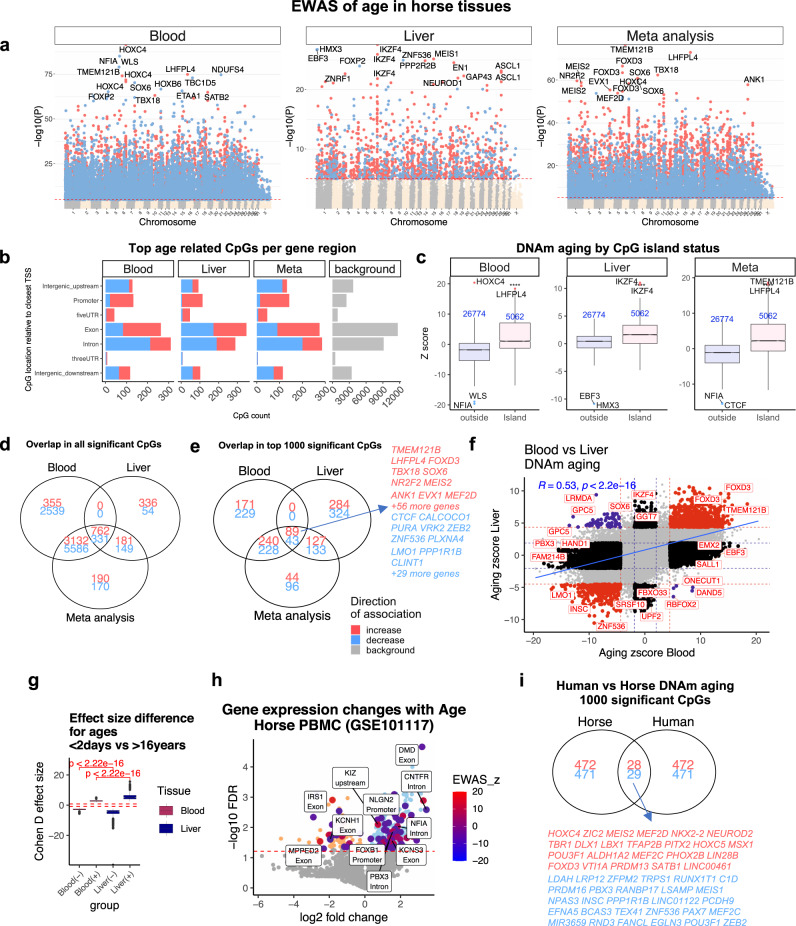
EWAS of age in horse blood and liver. Stouffer meta-analysis results between blood (*n* = 188) and liver (*n* = 48). **a** EWAS of age (Pearson correlation test) versus horse genome coordinates (Equus_caballus.EquCab3.0.100). Red dotted line corresponds to *p* = 10^−5^ (blood false discovery rate FDR < 2.5e−5, liver FDR < 0.00018, meta FDR < 3e−5). Significant CpGs are colored in red (age-related increase) and blue (decrease). Top 15 CpGs are labeled by neighboring genes. **b** Top CpGs in each tissue relative to adjacent transcriptional start sites. Gray corresponds to 31836 CpGs in the horse genome. **c** Box plot of age effects versus CpG island status. *Z* statistics resulted from applying the Fisher z-transformation to Pearson correlation coefficients. The numbers of CpGs are reported in blue text. The top four age-related CpGs in each tissue are labeled by adjacent genes. Boxes show the interquartile range (IQR) of the *Z* scores. The notches indicate the 95% confidence interval of the median. The whiskers represent 1.5*IQR length of the *Z* scores. Venn diagram of the overlap of (**d**) all significant CpGs, **e**) top 1000 (500 in each direction) significant CpGs. Significance thresholds: blood, *p* < 9.2e−27 (FDR < 2.6e−25); liver, *p* < 6.9e−6 (FDR < 1.3e−4); meta-analysis, *p* < 1.2e−20 (FDR < 2.4e−19). **f** Age effects in blood versus liver. Red dotted line: *p* < 10^−4^; blue dotted line: *p* > 0.05; Red dots: shared CpGs; blue dots: CpGs whose age correlation differs between blood and liver tissue. R: Pearson correlation coefficient. **g** Effect size (Cohen’s D) of age group (<2 days versus >16 years). Top 500 CpGs that gain methylation (denoted by +) and top 500 CpGs that lose methylation (denoted −). Red dashed line corresponds to Cohen *d* = |0.8|. Kruskal–Wallis test for tissue comparison. **h** Age-related mRNA changes in horse blood^32^ (GSE101117). Log (base 10) transformed FDR (*y*-axis) based on linear regression. The large blue and red dots report genes with at least one CpG that change with age in horse blood methylation data (Supplementary Data 3). **i** Venn diagram of the top 1000 (500 per direction) significant age-related CpGs in the blood of horses and humans (human *n* = 267, aged between 12 and 68).

The mammalian methylation array contains 31,836 probes that could be aligned to specific loci adjacent to 5093 unique genes (~17% coverage on 29,133 genes) in the horse (*Equus caballus* EquCab3.0.100) genome. Our epigenome-wide association studies (EWAS) correlated each of these CpGs with chronological age in horse blood (*n* = 188) and liver (*n* = 48) samples. The top DNA methylation changes in each tissue are as follows: blood, *HOXC4* intron (*z* = 20), and *NFIA* intron (*z* = −19); and liver, *IKZF4* exon (*z* = 11), and upstream of *HMX3* (*z* = −10) (Fig. 3a; Supplementary Data 1). Tissue level meta-analysis identified 10,501 CpGs with large age-related methylation changes in both blood and liver. Some of these include increased methylation of cytosines close to *TMEM121B*, *LHFPL4,* and *FOXD3* exons and *TBX18* promoter regions (Fig. S5).

At a nominal *p*-value < 10^−5^, 12,705 (FDR < 2.5 × 10^−5^) and 1813 (FDR < 0.0001) CpGs were found to be related to age in blood and liver, respectively (Fig. 3d). The discrepant number of significant age-related CpGs in each tissue probably reflects differences in sample size (*n* = 188 blood samples versus 48 liver samples, Table 1). To remove the bias resulting from differences in sample sizes and focus on CpGs with the strongest age effects, we also report results for the top 500 positively and top 500 negatively age-related CpGs in each tissue (Fig. 3f).

Age-related CpGs were found to be located in all genic and intergenic regions that can be defined relative to transcriptional start sites, which mirrors the distribution of the CpGs on the mammalian array (Fig. 3b). Further, CpGs located in CpG islands showed a higher correlation with age than non-island CpGs in horse tissues (Kruskal–Wallis *p* < 10^−22^, Fig. 3c). Aging effects in horse blood are positively correlated with those in horse liver (*r* = 0.53, *p* < 10^−16^, Fig. 3d, f). A Venn diagram reveals that the top 500 positively age-related CpGs in blood and liver share 89 CpGs in common (Fig. 3e). The top 500 negatively age-related CpGs in blood and liver share fewer CpGs (43).

We observe strong effect sizes for the top 500 negatively age-related CpGs in blood and liver: Pearson correlation coefficients between CpGs and age range from *R* = −0.894 to *R* = −0.656 in blood and from *R* = −0.92 to −0.58 in liver (Supplementary Data 1). Similarly, large effect sizes can also be observed for the top 500 positively age-related CpGs in blood and liver: R ranges from 0.670 to 0.904 in blood and from 0.676 to 0.926 in the liver (Supplementary Data 1). An alternative measure of effect size was obtained by our 2 group comparison between newborns and horses older than 16 (Fig. 3g). According to the standardized difference in methylation, Cohen’s *D* statistic, age has a stronger effect on liver than on blood (Kruskal–Wallis test *p* < 10^−16^, Fig. 3g).

We analyzed gene set enrichment of the top 500 positively and top 500 negatively age CpGs in each tissue with the GREAT software^26^. The significance thresholds for these top CpGs are as follows: blood, *p* < 9.2 × 10^−27^ (FDR < 2.6 × 10^−25^); liver, *p* < 6.9 × 10^−6^ (FDR < 1.3 × 10^−4^); meta-analysis, *p* < 1.2 × 10^−20^ (FDR < 2.4 × 10^−19^). To remove any bias resulting from the design of the mammalian array platform, we specified CpGs located on the mammalian array as the background set. Top 500 CpGs with a significant positive age correlation in horse blood and/or liver are proximal to genes that play a role in developmental processes (hypergeometric *p* < 10^−50^, gene ontology identifier GO:0032502) and multicellular organism development (*p* < 10^−50^, GO:0035264, Fig. S6A). The implicated genes can be found in Supplementary Data 2. Further, these CpGs are enriched for genes that give rise to mouse phenotypes related to development, such as “lethality during fetal growth through weaning”, “preweaning lethality” (*p* < 10^−50^, Fig. S6B)^26,27^. Finally, these positively age-related CpGs were located in gene regions targeted by polycomb repressor complex 2 (e.g., EED, SUZ12) targets that are marked with H3K27ME3 modification (*p* < 10^−50^, Fig. S6D). Overall, these results from this equine EWAS of age are consistent with those in humans and other mammalian species^28–31^.

Although a large number of CpGs were changed by age in both liver and blood, there were several that were unique to each tissue. In particular, 26 CpGs exhibited a divergent aging pattern between these two tissues (Figure S5). For example, while methylation in *GPC5* exon-1 is decreased with age in blood, it is increased in horse liver.

We studied the overlap between age effects in horse blood with those in human blood. A subset of 57 CpGs were shared between the top age-related CpGs in these species (Fig. 3I). This shows that DNAm aging between these species is partially converged, and why a dual-species epigenetic clock can be developed for horses and humans.

### Transcriptomic data in horses

We intersected genes implicated by our EWAS of age in horse blood with age-related mRNA changes implicated by published peripheral blood mononuclear cell (PBMC) transcriptomic data in horses (*n* = 12)^32^. In total, 322 genes (243 upregulated, 79 downregulated) were differentially expressed at 5% FDR by age in horse PBMC (Fig. 3h). We observed nominally significant overlap (hypergeometric *p* = 0.033, Odds ratio = 1.4) between age effects on mRNA and age effects on DNAm levels at these genes. A subset of 79 out of 322 genes had at least one CpG with age-related methylation change in our blood data, including *NFIA*, *DMD*, *IRS1*, and *CNTFR*. Supplementary Data 3 reports all the significant CpGs and their corresponding mRNA changes with age in the PBMC of horses.

### Effect of castration

Castration is a common practice. At puberty, which occurs between 12–15 months of age in horses, the concentrations of testosterone and estrone sulfate in intact stallions are ten and 100-fold greater, respectively, than in geldings^33–35^. Thus, castration presents an opportunity to test whether sex hormones affect the rate of epigenetic aging in male horses. This has important implications for cancer risk in horses, as castrated males were previously shown to be at a higher risk for ocular squamous cell carcinoma than females or stallions^36–39^. We employed the horse clocks to study whether castration affected epigenetic aging rates. Our primary analysis focused only on male samples. We evaluated the effect of castration on the epigenetic age of blood and liver. Multivariate regression models that regressed leave-one-out estimates did not show a significant association between castration and aging, irrespective of the age stratum. Our multivariate analysis based on leave-one-sample out estimates has an obvious limitation: both castrated and intact animals were used in the training set, which may condition out the effect of castration. Therefore, we repeated the analysis by developing a clock with female samples (training data) and applied it to male samples (test data). Again, we did not find a significant association of castration on epigenetic aging in blood. After failing to observe any significant association, we carried out secondary analyses in different age groups. We did not detect a significant association for castration in any age group. For example, we did not find significant associations in males older than five years or males younger than 15 years. This negative result echoes the same finding for blood samples from cats^29^. By contrast, castration was found to slow epigenetic aging in ear samples from sheep^40^.

**Fig. 4:**
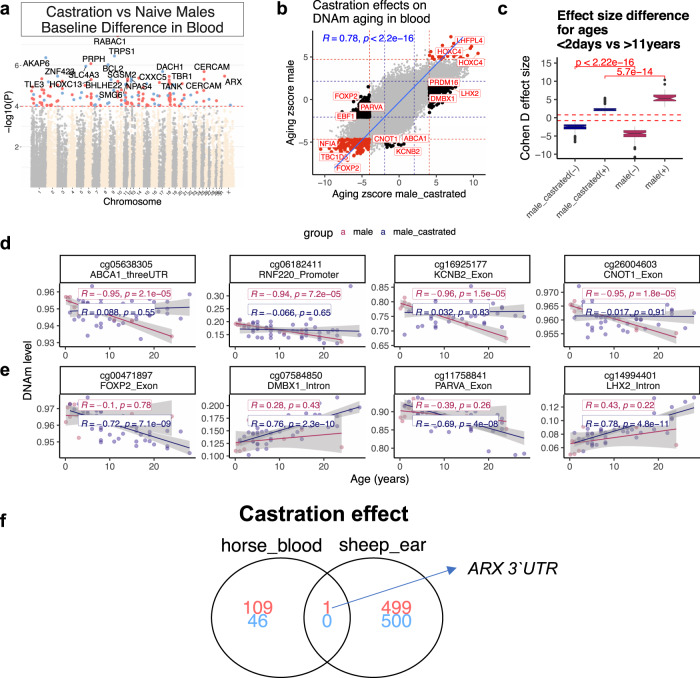
Castration moderately alters DNAm profile of horse blood. **a** Manhattan plots of the EWAS of castration, in the blood of male horses. Statistics: Multivariate linear regression model whose dependent variables are CpGs and whose co-variates are castration status and chronological age. Sample size: geldings, 48; stallions, 10. The coordinates are estimated based on Equus_caballus.EquCab3.0.100 genome assembly. The direction of associations with *p* < 10^−4^ (FDR < 0.02, red dotted line) is highlighted by red (increase) and blue (decrease) colors. Top 15 CpGs (*p* < 4.7e−6, FDR < 0.008) were labeled by the neighboring genes. **b** Sector plot of aging effects on blood methylation levels by castration status in male horses. The Z statistics result from applying the Fisher z-transformation to the Pearson correlation between CpG and age. Red-dotted line: p < 10^−4^; blue-dotted line: *p* > 0.05; Red dots: age-related CpGs not affected by castration; black dots: CpGs whose aging pattern differs between geldings and stallions. **c** The effect size of age on DNAm is larger in blood of naïve vs the castrated male horses. The effect size is calculated by Cohen *D* method between age groups <2 days vs. >11 years horses. Only the top 1000 significant CpGs per tissue (500 per direction) are presented in the box plot. (+) and (−) indicate the direction of change for each group. The dashed red line indicates Cohen *d* > |0.8 | , which means a large effect size. **d**, **e** Scatter plots of selected CpGs that change with age only stallions (**d**), or geldings (**e**) blood. The red dots and blue dots in the scatter plot correspond to blood samples from geldings and stallions, respectively. The shading visualizes the 95% confidence band of the linear regression model. *R*: Pearson correlation coefficient. These relationships require validation in new data and also a consideration for a potential confounding effect of horse breeds. **f** The overlap of castration methylation signatures between horse blood and sheep ears^40^. Although we considered the top 1 thousand significant CpGs in sheep (500 in each direction), we only found one overlapping CpG.

Aging effects on CpG methylation in geldings correlated strongly with those of stallions (*r* = 0.78) (Fig. 4b). There were nevertheless a few loci with methylation levels that changed with age only in geldings. For example, a CpG in the exon of *FOXP2* has decreased methylation with age in geldings, while a CpG in the 3′UTR of *ABCA1* shows an increase of methylation only in stallions (Fig. 4d, e; Supplementary Data 4). According to Cohen’s D statistic, aging effects on methylation levels were stronger in stallions than geldings (Fig. 4c), suggesting a larger age-related methylation change in non-castrated animals. Pearson correlation coefficients for individual CpGs are reported in Supplementary Data 4. In general, there was a moderate difference between geldings and stallions with regards to the baseline mean methylation of CpGs in the blood (independent of age). Some of the top methylation signatures of geldings included an increase of methylation in *RABAC1* intron, *PRPH* exon, and a decrease of methylation in *TRPS1* intron and *AKAP6* intron (Fig. 4a**;** Supplementary Data 5). Castration-related genes, implicated by mean methylation differences or different aging patterns, were related to development of nervous system, cartilage, connective tissue, and muscle physiology (Fig. S7; Supplementary Data 6).

We used results from a recent study evaluating the effects of castration on DNAm patterns in ear punch samples from sheep^40^ to investigate if commonality exists between observed methylation loss or gain following castration from these two divergent species. EWAS comparisons of the top 500 age-related CpGs revealed a single CpGs (adjacent to *ARX* 3’UTR) that gained methylation following castration in both horse blood and sheep ears (Horse blood: *p* = 3.49e−6, false discovery rate = 0.008, sheep ear: *p* = 3.14e−17, FDR = 4.6e−15, Fig. 4f). The low overlap (single CpG near *ARX*) is probably due to a tissue difference between horse and sheep studies (blood versus ear) and species differences.

### DNAm relate to gene expression differences in horse tissues

**Fig. 5:**
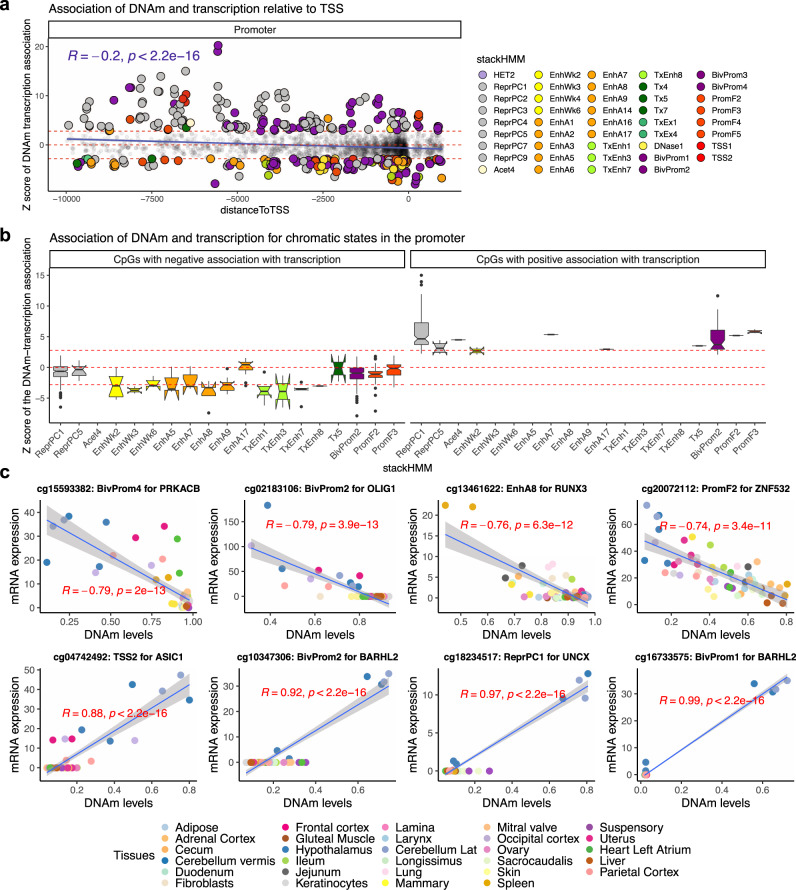
DNAm levels in promoters relate to gene expression changes across horse tissues. This analysis was based on a Pearson correlation of DNAm and mRNA level of the adjacent genes in 29 different tissues from two female horses. Each CpG was assigned to one gene based on the closest distance to the transcriptional start sites. **a**, **b** The *y*-axis reports a *Z* statistic of a correlation test between the methylation level of each CpG and gene expression of the adjacent gene across tissues. The *Z* statistics result from applying the Fisher z-transformation to the Pearson correlation between CpG and mRNA. **a** The *x*-axis reports the distance to the transcription start site. The analysis is limited to CpGs located in the promoter regions of genes. Genes are colored by chromatin states of their respective gene promoters. The chromatin states are based on the stackHMM annotations, which represent a consensus chromatin state in over 100 human tissues^68^. A description of the chromatin states is provided in Supplementary Data 7. Red horizontal lines correspond to significance threshold (*Z* > 2.8 and Z < −2.8 values, two-sided *p* < 0.005). **b** Boxplot of DNAm-mRNA association by stackHMM state in CpGs with the significant cis-expression relationship. Boxes show the interquartile range of the *z* scores (i.e. lower and upper 25^th^ percentile). The notches indicate the 95% confidence interval of the median. The whiskers represent 1.5*IQR length of the *z* scores. This analysis focuses on CpGs that are located in promoter and have a significant (Pearson correlation p < 0.05) DNAm-mRNA association with the adjacent gene. Thus, the CpGs in panel **b** is the subset of CpGs from panel a, namely those that have a significant mRNA-DNAm association. We found 256 CpGs with a positive association, 2223 CpGs with a negative association. To simplify the figure, we only reported the stackHMM states with a median DNAm-mRNA association of *z* > 2.8 or *z* < −2.8 (Pearson correlation *p* < 0.005). **c** Scatter plots of selected CpGs with DNAm-mRNA association in horse tissues. R: Pearson correlation coefficient. *P*: Two-sided Student *t*-test *p*-value. Het heterochromatin, ReprPC repressed by polycomb proteins, Acet acetylation, EnhWk weak enhancers, EnhA enhancers, TxEnh transcribed and enhancer, Tx transcription, TxEx exon, BivProm bivalent-promoter, Prom promoter, TSS transcriptional-start-site.

Meaningful interpretation of epigenetic findings requires the coupling of DNA methylation changes with those of gene expression. This challenge is further compounded by comparisons between tissues and species. Our study provides a rare opportunity to address this question by studying CpGs that are located in genomic regions that are conserved across mammalian species. Here, we integrated DNAm and RNA-seq data from 57 samples (originating from 29 different tissues of two horses^25^) to uncover the relationship between methylation changes of promoter CpGs with the expression of adjacent genes. Our analysis revealed that this relationship is dependent on the distance between the methylation site and the transcriptional start site (TSS) (Fig. 5a). In general, methylation of CpGs that are closer to TSSs (from 10,000 nucleotides downstream to 1000 upstream of TSS) has a stronger repressive effect on mRNA levels (*r* = −0.2, *p* < 2 × 10^−16^). This negative relationship was independent of CpG island status of the loci (Fig. S8).

Regulation of gene expression, however, is a multi-faceted process, of which methylation of the promoter is just one of the determinants. The chromatin context within which the CpGs are located is another feature that may exert a strong influence. As such, we first sought to ascertain the chromatin features within which the above CpGs are positioned, and then incorporate this information into the analysis of the impact of methylation of these CpGs on gene expression. Since chromatin states that are specific to the horse genome are presently unavailable, we used the “stacked chromatin states (stackHMM)” that identifies chromatin features based on the consensus of over 100 human cell types (Supplementary Data 7)^41^. Despite the species difference, this approach can nevertheless prove highly informative because the design of the mammalian methylation array was based on DNA loci that are conserved across mammals^41^, allowing chromatin features identified by stackHMM to be applied not only to the horse but other mammalian species as well. Interestingly, this analysis led to the observation that the contextual chromatin feature of CpGs was an even better indicator of the DNA methylation-gene expression relationship. In general, methylation of CpGs within enhancers appears to correlate with reduced gene expression (e.g. EnhWk2,3,6; EnhA3,6,7:9; and TxEnh1,3,7,8) (Fig. 5b, c). In contrast, increased gene expression is correlated with methylation of CpGs within polycomb repressed targets (e.g., ReprPC1–5), bivalent promoters (e.g., BivProm1–4), promoter flanks (e.g. PromF4,5), and transcriptional start sites (e.g., TSS1,2) (Fig. 5b, c). Correlations of individual CpG methylation with mRNA expression are reported in Supplementary Data 8. Since the cerebellum was an outlier for some of the CpG-mRNA associations, we examined if the relationship with chromatin states is sensitive to cerebellum inclusion. Our sensitivity analysis revealed that cerebellum did not affect our findings (Fig. S9), suggesting a converging pattern between chromatin states and DNAm-mRNA association in tissues.

## Discussion

As human methylation arrays (450K and EPIC) are specific to the human genome, their utility could not be extended to other species. A critical step toward crossing the species barrier was the employment of a mammalian DNA methylation array^41^, which led to the acquisition of the most comprehensive epigenetic dataset of domestic horses thus far. Using these data, we constructed six highly accurate DNA methylation-based age estimators for horses that are applicable to their entire life course (from birth to old age). The fact that the presented horse clocks are highly accurate according to unbiased cross-validation studies (Figs. 1 and 2) shows that one can build epigenetic clocks using CpGs that are embedded within evolutionarily conserved DNA sequences.

The same mammalian array was applied to species of particular interest: four equid species and humans. The resulting data facilitated the development of two noteworthy multi-species clocks: the human-horse dual-species clock and the equid clock that apply to blood samples from all equid species. Each of these multi-species clocks corresponds to a multivariate regression model with the same set of covariates, i.e., the same set of CpGs and coefficient values are being used. However, the dependent variable in the regression model adjusts for systematic differences in species characteristics such as maximum lifespan or age at sexual maturity. Relative age, defined as the ratio of chronological age to maximum lifespan, is used as a dependent variable of the human-horse clock. The mathematical operation of generating a ratio eliminates chronological unit of time and produces a value that indicates the age of the organism in respect to the maximum age of its own species. The equid clock uses a different age transformation that does not require knowledge of maximum lifespan. Rather, it uses average age at sexual maturity, which is a more robustly estimated species characteristic, in its log-linear transformation of age. Collectively, the ability to use a single mathematical formula to measure epigenetic age in different species and the replacement of the chronological unit of time with a transformed version are two significant innovations that will propel cross-species research. While this article focused on equids, we have described multi-species clocks for several other species and all mammals^29–31,42–44^. In general, we expect that species-specific clocks (pure horse clocks) will outperform pan-mammalian clocks because methylation levels are strongly affected by genetics and the environment.

The species characteristics used in our age transformations are debatable, e.g., the reported maximum lifespan of 57 years for horses will appear unrealistic to many experts. We respond to these concerns in two ways. First, we used species characteristics from a rigorous and highly regarded database, anAge^45^. Second, and more importantly, our mathematical models are highly robust with respect to different choices of these species’ characteristics. Similarly, accurate multi-species clocks would result when using alternative choices of maximum lifespan. Our horse data are limited with respect to the upper limit of the ascertained ages (29 years).

Beyond utilizing the horse methylation data sets to develop epigenetic clocks, we also investigated the characteristics of age-related CpGs. Many CpGs with a high positive age correlation in horse liver showed the same in horse blood and vice versa. Negatively, age-related CpGs were less conserved across tissue types. An unbiased functional enrichment study demonstrates that positively age-related CpGs are adjacent to genes that play a role in development (gene ontology category of development, Fig. S6). The role of development is also suggested by enrichment of positively age-related CpGs with respect to bivalent chromatin domains and targets of polycomb repressive complex 2. Thus, the connection between development and aging, albeit not immediately intuitive, is difficult to ignore. For example, we find a positive correlation between age effects in human blood and those in horse blood (Fig. 3I). Overall, the results of our EWAS of age in horses echo those in humans and many other mammalian species^2,4,46^. A prior methylation study in horse leukocytes based on a different genomic platform (Reduced Representation Bisulfite Sequencing) found increased methylation near *IGFR1*^47^. Corroborating these results, we found a CpG in an exon of *IGF1R* with an age-related gain of methylation in horse blood. We show that age-associated EWAS hits in horse blood are adjacent to genes implicated by a transcriptomic study of aging effects in horse blood-related mRNA changes^32^.

It would indeed be very informative to resolve a direct relationship between DNAm and gene expression changes. This has remained one of the challenging and limiting features of understanding DNA methylation changes because data for gene expression is often unavailable. Fortuitously, such data for these tissues are available in the horse tissue atlas (but not in our aging study). The tissue atlas allowed us to correlate DNAm and transcriptional data across 29 tissues from the horse tissue atlas. Our analysis suggests that cytosine methylation alone has a modest correlation with gene expression outcomes. However, the incorporation of contextual chromatin elements (enhancers, promoters, etc.) to the analysis increased the magnitude of the correlation between CpG methylation and gene expression. Specifically, methylation of CpGs within enhancers is more likely to correlate with reduced gene expression, while methylation of CpGs in polycomb repressed targets, bivalent promoter, promoter flanks, and transcriptional start sites result in largely increased gene expression (Fig. 5a, b). This may at first appear counter-intuitive, as methylation of promoters is often associated with repression of transcription. It is to be noted, however, that this notion is largely true for promoters with adjacent CpG islands. In the specific case of PRC targets and bivalent chromatin domains, our analysis is consistent with the recent observation that an increase of methylation in bivalent chromatin domains results in a reduced presence of the repressive histone H3K27me3, causing the balance of histone ratio towards the transcription-promoting H3K4me3^48^. This is consistent with the finding that DNA methylated regions of the genome are largely low in or devoid of H3K27me3, possibly due to the unfavorable binding of PRC2, which is required for methylation of H3K27 histone. Interestingly, methylation of bivalent chromatin domains was reported to correlate with increased expression of developmental genes^49^, which incidentally are the predominant genes proximal to age-related CpGs. Our chromatin state analysis was limited due to its use of human cell lines. A horse-specific chromatin state annotation is expected to become available as part of the ongoing FAANG initiative^25^.

The association between CpG methylation level, genomic elements, and gene expression is a valuable tool to interpret methylation array findings from all mammalian species. For example, our analyses of age-related methylation changes in horse tissues reveal that CpGs that became increasingly methylated with age are located largely within promoters and CpG islands. Two of the top CpGs that exhibited increased methylation with age in horses were located in the promoters of *TBX18* and *FOXD3*. However, while the *TBX18* promoter had a strong negative correlation with DNAm-mRNA (*z* = −2.8), the *FOXD3* promoter showed a positive DNAm–mRNA correlation in horse tissues (*z* = 1.5). Thus, we can deduce that *TBX18* expression decreases with age, but *FOXD3* mRNA levels will increase with age. Cross-species expansion of this finding is only possible due to the design of the mammalian methylation array for highly conserved genomic regions^41^. Thus, this multi-omics analysis of horse tissues is a tool to link DNAm with transcription in other studies based on the mammalian methylation array. Such a link is essential for functional interpretation of the findings and also for the experimental design of gene-perturbation studies.

When it comes to aging effects, we did not have access to transcriptomic data and methylation data from the same animals. Based on human studies, we expect that the cis relationship between methylation and gene expression will be weak in blood due to large cell-to-cell variability^50^. Recent studies reveal stronger relationships between methylation levels and transcriptomic changes in single-cell data^51–53^.

The presented horse clocks lend themselves to estimating the chronological age of any animal. Experience with human clocks suggests that epigenetic age estimates may be affected by technical issues and biological differences, such as diet, viral infections, environmental factors, and even genetic differences that could result in an offset, i.e., a constant difference between epigenetic age estimate and the true chronological age. Future research is needed to determine whether the discrepancy between epigenetic age and chronological age relates to pathologies such as cancer or other age-related conditions in horses.

Future studies could develop second-generation epigenetic clocks that relate to mortality/morbidity risk similar to what has been achieved in humans^4,19,54^.

## Methods

### Ethics

This research complied with all relevant ethical regulations overseen by 4 ethics review boards:

This horse tissue collection protocol was approved by the UC Davis Institutional Animal Care and Use Committee (Protocol#19037).

Zebra samples were collected under a protocol approved by the Research Safety and Animal Welfare Administration, University of California Los Angeles: ARC # 2009-090-31, originally approved in 2009.

The human skin samples were acquired with informed consent prior to collection of human skin samples approved by the Oxford Research Ethics Committee in the UK; reference 10/H0605/1. Participants were not compensated. The secondary use of the other de-identified/coded human tissue samples (blood, postmortem tissues) is not interpreted as human subjects research under U.S. Department of Health & Human Services 45 CFR 46. Therefore, the need to obtain written, informed consent from human study participants was waived (secondary use of de-identified tissues). Human samples were covered by University of California Los Angeles IRB#18-000315.

#### Study samples

##### Horses

We generated DNA methylation data from *n* = 42 different horse tissues collected at necropsy (Table 1). The tissue atlas was generated from two Thoroughbred mares as part of the FAANG initiative^25^, with the following tissues profiled: adipose (gluteal), adrenal cortex, blood (PBMCs; only *n* = 1 mare), cartilage, cecum, cerebellum (2 samples each from lateral hemisphere and vermis), frontal cortex, duodenum, fibroblast, heart (2 samples each from the right atrium, left atrium, right ventricle, left ventricle), hypothalamus, ileum, jejunum, keratinocyte, kidney (kidney cortex and medulla), lamina, larynx (i.e., cricoarytenoideus dorsalis muscle), liver, lung, mammary gland, the mitral valve of the heart, skeletal muscle (gluteal muscle and longissimus muscle), occipital cortex, ovary, parietal cortex, pituitary, sacrocaudalis dorsalis muscle, skin, spinal cord (C1 and T8), spleen, suspensory ligament, temporal cortex, tendon (deep digital flexor tendon and superficial digital flexor tendon), uterus^25^. These tissues were also used for RNA-seq analyses.

Blood samples were collected via venipuncture into EDTA tubes from across 24 different horse breeds (buffy coat). Most of the samples were from the Thoroughbred (TB) (*n* = 79) and American Quarter Horse breeds (QH, *n* = 62). For the following breeds, we had between one and six blood samples: Andalusian, Appaloosa, Arabian, Dutch Warmblood, Hanoverian, Holsteiner, Irish Sport Horse, Lipizzaner, Lusitano, mixed breed, Oldenburg, Paint or Paint cross, Percheron, Shire, Standardbred, Warmblood and Welsh Pony. The *n* = 49 liver samples originated from necropsy collections of horses across 19 different breeds, with most of the liver samples from QHs (*n* = 20). All collection protocols were approved by the UC Davis Institutional Animal Care and Use Committee (Protocols #20751 and 21455, respectively).

### Additional equid species

The data from the three additional equid species are described in a companion paper^55^ that focuses on plains zebras (*Equus quagga*). Briefly, both blood (*n* = 76 including 42 female samples, aged between 0.16 and 20.2 years, mean age = 5.2 years) and skin biopsy (*n* = 24 including 9 female samples, aged between 0.16 and 24.8 years, mean age=5.9 years) samples from plains zebras were obtained from a captive population of zebras maintained in a semi-wild state by the Quagga Project^56^ in the Western Cape of South Africa. The population was founded in 1989 by 19 individuals (9 from Etosha National Park in Namibia, 10 from the Kwazulu-Natal in South Africa). Skin samples were taken by remote biopsy dart (1 mm wide by 20-25 mm deep plug) and preserved in RNAlater (Qiagen). Blood samples were taken opportunistically during veterinarian visits and preserved in EDTA tubes. Most samples were collected from different individuals, except for two animals that were sampled twice some years apart. All samples were stored at -20 °C. After eliminating samples with low confidence for individual identity and age, we retained 76 blood samples and 20 skin samples. We retained the founder, however, in an effort to extend the age range represented in the skin clock.

Blood samples from Grevy’s zebra (total *n* = 5 comprised of 4 males and 1 female, age ranged from 2.3 years to 18.5 years) and Somali wild ass (total *n* = 7 comprised of 6 males and 1 female, age ranged from 2.4 years to 10.0 years) were opportunistically collected from zoo-based animals during routine health exams^31,55^.

### Human tissue samples

To construct the human-horse clock, we analyzed the generated methylation data from *n* = 1,352 human tissue samples (including adipose, blood, bone marrow, dermis, epidermis, heart, keratinocytes, fibroblasts, kidney, liver, lung, lymph node, muscle, pituitary, skin, spleen) from individuals whose ages ranged from 0 to 101. Of the *n* = 1352 tissues, *n* = 655 came from women (Supplementary Data 9). These human tissue samples came from multiple sources: tissue and organ samples from the National NeuroAIDS Tissue Consortium^57^, blood samples from the Cape Town Adolescent Antiretroviral Cohort study^58^, and blood, skin, and other primary cells provided by Kenneth Raj^59^ and blood samples from the PEG study^60^.

#### DNA methylation data

The mammalian DNA methylation data were generated using the mammalian methylation array (HorvathMammalMethylChip40) based on 37492 CpG sites^41^. Not all of these CpGs apply to horses. Here we focused on 31,836 CpGs that could be mapped to the horse genome (Equus_caballus.EquCab3.0.100, https://www.ncbi.nlm.nih.gov/assembly/GCF_002863925.1/).

Thus, the mammalian array covers relatively few cytosines in the horse genome. In particular, it does not cover horse-specific cytosines.

Genome coordinates for each CpG are provided on the GitHub page of the Mammalian Methylation Consortium^61^; see the section on data availability. The manifest file of the mammalian methylation array can be found at Gene Expression Omnibus (GEO) at NCBI as platform GPL28271. The SeSaMe normalization method was used to define beta values for each probe^62^.

#### RNA-seq data

Strand-specific RNA libraries were created following poly-A selection. Libraries were sequenced at 2x150bp on Illumina HiSeq2500, with a targeted depth of 30 million reads. RNA-seq data were used to quantify transcripts annotated in Ensemble annotation (GCA_002863925.1, release 103) using Salmon mapping-based mode^63^.

#### Penalized regression models

Penalized regression models were created with glmnet^64^. We investigated models produced by “elastic net” regression (alpha = 0.5). The optimal penalty parameters in all cases were determined automatically by using a 10 fold internal cross-validation (cv.glmnet) on the training set. By definition, the alpha value for the elastic net regression was set to 0.5 (midpoint between Ridge and Lasso type regression) and was not optimized for model performance.

We performed a cross-validation scheme for arriving at unbiased (or at least less biased) estimates of the accuracy of the different DNAm based age estimators. One type consisted of leaving out a single sample (LOOCV) from the regression, predicting an age for that sample, and iterating over all samples. A critical step is the transformation of chronological age (the dependent variable). While no transformation was used for the blood clock for horses, we did use a log-linear transformation for the dual-species clock of chronological age (Supplement). Details on the clocks (CpGs, genome coordinates), coefficient values, and age transformations are provided in the Supplement.

#### Relative age estimation

Relative age estimation was performed to introduce biological meaning into the age estimates of horses and humans, which have very different lifespans. Additionally, this estimation serves to overcome the inevitable skewing due to the unequal distribution of data points from horses and humans across the age range. Relative age estimations were calculated using the formula: Relative age = Age/maxLifespan, where the maximum lifespan for the two species was chosen from the “anAge” database (57 for horses and 122.5 for humans^45^).

### Maximum lifespan of horses

The maximum age for horses (57 years) will sound too high for many experts. Miniature horses appear to live longer, however, there are no miniature horses in our dataset. The anAge database^45,65^ record for horse states the following. Quote “One Icelandic … horse…is reported to have lived 57 years (Richard Miller, pers. comm.). Anecdotal evidence tells of a horse…that lived for 62 years in England, but that record is unverified.” A news article on the Icelandic horse “Tulle” can be found online (Horse Tulle URL). The oldest regular-sized horse with a well-documented age appears to have reached an age of 53 according to the Official Guide for Determining the Age of the Horse from the American Association of Equine Practitioners (B. Wright 1999 URL).

#### Epigenome-wide association studies of age

EWAS was performed in each tissue separately using the R function “standardScreeningNumericTrait” from the “WGCNA” R package^66^. Next, the results were combined across tissues using Stouffer’s meta-analysis method.

#### GREAT analysis

We analyzed gene set enrichments using GREAT^26^. The GREAT enrichment analysis automatically conditioned out (removed) any bias resulting from the design of the mammalian array by using a background set of CpGs that map to horses and are located on the mammalian array. Thus, our GREAT enrichment analysis conditioned out (removed) any bias resulting from restricting the analysis to conserved CpGs on the mammalian array platform. The GREAT software performs both a binomial test (over genomic regions) and a hypergeometric test over genes.

We performed the enrichment based on default settings (Proximal: 50.0 kb upstream, 1.0 kb downstream, plus Distal: up to 1000 kb) for gene sets implemented in GREAT. To avoid large numbers of multiple comparisons, we restricted the analysis to the gene sets whose sizes ranged from 10 to 3000 genes. We report one-sided nominal *P* values and two adjustments for multiple comparisons: Bonferroni correction and the Benjamini-–Hochberg false discovery rate”.

#### Tissue atlas: correlating DNAm with mRNA

The annotation file of the mammalian methylation array provides the genomic location of all target CpGs relative to the adjacent transcriptional start site in the horse genome^41^. Thus, we could link each CpG with mRNA level of the adjacent gene. We assigned each CpG to the gene whose transcriptional start site is closest to the CpG. A detailed description of alignment and gene assignment is reported in our mammalian array reference paper^41^. The R function “corAndPvalue” from the “WGCNA” R package^66^ was used to calculate Pearson correlation coefficients, its Fisher transformation, and 2 sided p values based on the Student *T* test.

### URL

B. Wright (1999) Official Guide for Determining the Age of the Horse, American Association of Equine Practitioners. http://www.omafra.gov.on.ca/english/livestock/horses/facts/info_age.htm Horse Tulle https://www.angelfire.com/az/testryder/home.html.

### Reporting summary

Further information on research design is available in the Nature Research Reporting Summary linked to this article.

## Supplementary information

Supplementary Information

Description of Additional Supplementary Files

Supplementary Data 1-10

Reporting Summary

## Data Availability

The methylation data from horses, zebras, and equids generated in this study have been deposited in Gene Expression Omnibus (accession numbers GSE174767, GSE184222, GSE184223). The RNA-seq data can be downloaded from https://www.ebi.ac.uk/ena/data/view/ERA1487553 The human methylation data were not generated for this study. These data will be presented in other publications^67^ and can be requested from SH. In addition, the data will be posted on GEO as part of the data release from the Mammalian Methylation Array Consortium. The mammalian methylation array is available through the non-profit Epigenetic Clock Development Foundation (https://clockfoundation.org/). We used species characteristics from the AnAge Database https://genomics.senescence.info/species/.

## Code availability

Details on the clocks (CpGs, genome coordinates) and R software code are provided in Supplementary Note 1 and in Supplementary Data 10. Genome annotations of the CpGs can be found on Github^61^ https://github.com/shorvath/MammalianMethylationConsortium/tree/v1.0.0.

## References

[1] Rakyan VK (2010). Human aging-associated DNA hypermethylation occurs preferentially at bivalent chromatin domains. Genome Res..

[2] Teschendorff AE (2010). Age-dependent DNA methylation of genes that are suppressed in stem cells is a hallmark of cancer. Genome Res..

[3] Issa J-P (2014). Aging and epigenetic drift: a vicious cycle. J. Clin. Investig..

[4] Horvath, S. & Raj, K. DNA methylation-based biomarkers and the epigenetic clock theory of ageing. *Nat. Rev. Genet*. 10.1038/s41576-018-0004-3 (2018). 10.1038/s41576-018-0004-3 29643443

[5] Field AE (2018). DNA methylation clocks in aging: categories, causes, and consequences. Mol. Cell.

[6] Bell CG (2019). DNA methylation aging clocks: challenges and recommendations. Genome Biol..

[7] Horvath S (2013). DNA methylation age of human tissues and cell types. Genome Biol..

[8] Petkovich DA (2017). Using DNA methylation profiling to evaluate biological age and longevity interventions. Cell Metab..

[9] Cole JJ (2017). Diverse interventions that extend mouse lifespan suppress shared age-associated epigenetic changes at critical gene regulatory regions. Genome Biol..

[10] Wang T (2017). Epigenetic aging signatures in mice livers are slowed by dwarfism, calorie restriction and rapamycin treatment. Genome Biol..

[11] Stubbs TM (2017). Multi-tissue DNA methylation age predictor in mouse. Genome Biol..

[12] Thompson MJ (2018). A multi-tissue full lifespan epigenetic clock for mice. Aging.

[13] Meer MV, Podolskiy DI, Tyshkovskiy A, Gladyshev VN (2018). A whole lifespan mouse multi-tissue DNA methylation clock. eLife.

[14] Marioni R (2015). DNA methylation age of blood predicts all-cause mortality in later life. Genome Biol..

[15] Christiansen L (2016). DNA methylation age is associated with mortality in a longitudinal Danish twin study. Aging Cell.

[16] Perna L (2016). Epigenetic age acceleration predicts cancer, cardiovascular, and all-cause mortality in a German case cohort. Clin. Epigenet..

[17] Chen BH (2016). DNA methylation-based measures of biological age: meta-analysis predicting time to death. Aging.

[18] Horvath S (2015). Decreased epigenetic age of PBMCs from Italian semi-supercentenarians and their offspring. Aging.

[19] Lu AT (2019). DNA methylation GrimAge strongly predicts lifespan and healthspan. Aging.

[20] Jylhava J, Pedersen NL, Hagg S (2017). Biological age predictors. EBioMedicine.

[21] Li X (2020). Longitudinal trajectories, correlations and mortality associations of nine biological ages across 20-years follow-up. eLife.

[22] Ferrucci L (2020). Measuring biological aging in humans: a quest. Aging Cell.

[23] Raj, K. & Horvath, S. Current perspectives on the cellular and molecular features of epigenetic ageing. *Exp. Biol. Med.*10.1177/1535370220918329 (2020). 10.1177/1535370220918329 PMC7787550 32276545

[24] Fahy GM (2019). Reversal of epigenetic aging and immunosenescent trends in humans. Aging Cell.

[25] Burns EN (2018). Generation of an equine biobank to be used for Functional Annotation of Animal Genomes project. Anim. Genet..

[26] McLean, C. Y. et al. GREAT improves functional interpretation of cis-regulatory regions. *Nat. Biotechnol.* **28**, 10.1038/nbt.1630 (2010). 10.1038/nbt.1630 PMC4840234 20436461

[27] Eppig JT, Blake JA, Bult CJ, Kadin JA, Richardson JE (2015). The Mouse Genome Database (MGD): facilitating mouse as a model for human biology and disease. Nucleic Acids Res..

[28] Robeck TR (2021). Multi-species and multi-tissue methylation clocks for age estimation in toothed whales and dolphins. Commun. Biol..

[29] Raj, K. et al. Epigenetic clock and methylation studies in cats. *GeroScience*10.1007/s11357-021-00445-8 (2021). 10.1007/s11357-021-00445-8 PMC8599556 34463900

[30] Prado NA (2021). Epigenetic clock and methylation studies in elephants. Aging Cell.

[31] Lu, A. T. et al. Universal DNA methylation age across mammalian tissues. Preprint at *bioRxiv*10.1101/2021.01.18.426733 (2021).

[32] Tallmadge RL, Wang M, Sun Q, Felippe MJB (2018). Transcriptome analysis of immune genes in peripheral blood mononuclear cells of young foals and adult horses. PLoS ONE.

[33] Arighi, M., Bosu, W. & Raeside, J. In *Proceedings of the 31st Annual Convention of the American Association of Equine Practitioners* (ed. American Association of Equine Practioners) 591–602 (1985).

[34] Arighi M, Bosu WT (1989). Comparison of hormonal methods for diagnosis of cryptorchidism in horses. J. Equine Vet. Sci..

[35] Cox JE (1989). Testosterone concentrations in normal and cryptorchid horses. Response to human chorionic gonadotrophin. Anim. Reprod. Sci..

[36] Schaffer PA, Wobeser B, Martin LE, Dennis MM, Duncan CG (2013). Cutaneous neoplastic lesions of equids in the central United States and Canada: 3,351 biopsy specimens from 3,272 equids (2000–2010). J. Am. Vet. Med. Assoc..

[37] Kafarnik C, Rawlings M, Dubielzig RR (2009). Corneal stromal invasive squamous cell carcinoma: a retrospective morphological description in 10 horses. Vet. Ophthalmol..

[38] Mosunic CB (2004). Effects of treatment with and without adjuvant radiation therapy on recurrence of ocular and adnexal squamous cell carcinoma in horses: 157 cases (1985–2002). J. Am. Vet. Med. Assoc..

[39] Michau TM, Davidson MG, Gilger BC (2012). Carbon dioxide laser photoablation adjunctive therapy following superficial lamellar keratectomy and bulbar conjunctivectomy for the treatment of corneolimbal squamous cell carcinoma in horses: a review of 24 cases. Vet. Ophthalmol..

[40] Sugrue VJ (2021). Castration delays epigenetic aging and feminizes DNA methylation at androgen-regulated loci. eLife.

[41] Arneson, A. et al. A mammalian methylation array for profiling methylation levels at conserved sequences. Preprint at *bioRxiv*10.1101/2021.01.07.425637 (2021). 10.1038/s41467-022-28355-z PMC8831611 35145108

[42] Horvath, S. et al. DNA methylation age analysis of rapamycin in common marmosets. *GeroScience*10.1007/s11357-021-00438-7 (2021). 10.1007/s11357-021-00438-7 PMC8599537 34482522

[43] Horvath, S. et al. Epigenetic clock and methylation studies in the rhesus macaque. *GeroScience*10.1007/s11357-021-00429-8 (2021). 10.1007/s11357-021-00429-8 PMC8599607 34487267

[44] Schachtschneider, K. M. et al. Epigenetic clock and DNA methylation analysis of porcine models of aging and obesity. *GeroScience*10.1007/s11357-021-00439-6 (2021). 10.1007/s11357-021-00439-6 PMC8599541 34523051

[45] de Magalhaes JP, Costa J, Church GM (2007). An analysis of the relationship between metabolism, developmental schedules, and longevity using phylogenetic independent contrasts. J. Gerontol. A Biol. Sci. Med. Sci..

[46] Thompson MJ, vonHoldt B, Horvath S, Pellegrini M (2017). An epigenetic aging clock for dogs and wolves. Aging.

[47] Zabek T (2019). Methylation marks of blood leukocytes of native hucul mares differentiated in age. Int. J. Genom..

[48] Dunican DS (2020). Bivalent promoter hypermethylation in cancer is linked to the H327me3/H3K4me3 ratio in embryonic stem cells. BMC Biol..

[49] Bernhart SH (2016). Changes of bivalent chromatin coincide with increased expression of developmental genes in cancer. Sci. Rep..

[50] van Eijk K (2012). Genetic analysis of DNA methylation and gene expression levels in whole blood of healthy human subjects. BMC Genom..

[51] Clark SJ, Lee HJ, Smallwood SA, Kelsey G, Reik W (2016). Single-cell epigenomics: powerful new methods for understanding gene regulation and cell identity. Genome Biol..

[52] Hu Y (2016). Simultaneous profiling of transcriptome and DNA methylome from a single cell. Genome Biol..

[53] Linker SM (2019). Combined single-cell profiling of expression and DNA methylation reveals splicing regulation and heterogeneity. Genome Biol..

[54] Levine, M. E. et al. An epigenetic biomarker of aging for lifespan and healthspan. *Aging (Albany NY)*10.18632/aging.101414 (2018). 10.18632/aging.101414 PMC5940111 29676998

[55] Larison B (2021). Epigenetic models predict age and aging in plains zebras and other equids. Commun Bio..

[56] Harley EH, Knight MH, Lardner C, Wooding B, Gregor M (2009). The Quagga project: progress over 20 years of selective breeding. Afr. J. Wildl. Res..

[57] Morgello S (2001). The National NeuroAIDS Tissue Consortium: a new paradigm in brain banking with an emphasis on infectious disease. Neuropathol. Appl. Neurobiol..

[58] Horvath S (2018). Perinatally acquired HIV infection accelerates epigenetic aging in South African adolescents. AIDS.

[59] Kabacik S, Horvath S, Cohen H, Raj K (2018). Epigenetic ageing is distinct from senescence-mediated ageing and is not prevented by telomerase expression. Aging.

[60] Horvath S, Ritz BR (2015). Increased epigenetic age and granulocyte counts in the blood of Parkinson’s disease patients. Aging.

[61] Horvath, S. & Haghani, A. 10.5281/zenodo.5711978. *Mammalian Methylation Consortium Github* https://github.com/shorvath/MammalianMethylationConsortium/tree/v1.0.0 (2021).

[62] Zhou W, Triche TJ, Laird PW, Shen H (2018). SeSAMe: reducing artifactual detection of DNA methylation by Infinium BeadChips in genomic deletions. Nucleic Acids Res..

[63] Patro R, Duggal G, Love MI, Irizarry RA, Kingsford C (2017). Salmon provides fast and bias-aware quantification of transcript expression. Nat. Methods.

[64] Friedman J, Hastie T, Tibshirani R (2010). Regularization paths for generalized linear models via coordinate descent. J. Stat. Softw..

[65] de Magalhaes JP, Costa J, Toussaint O (2005). HAGR: the Human Ageing Genomic Resources. Nucleic Acids Res..

[66] Langfelder P, Horvath S (2008). WGCNA: an R package for weighted correlation network analysis. BMC Bioinforma..

[67] Horvath, S. et al. Pan-primate DNA methylation clocks. Preprint at *bioRxiv*10.1101/2020.11.29.402891 (2021).

[68] Vu, H. & Ernst, J. Universal annotation of the human genome through integration of over a thousand epigenomic datasets. Preprint at *bioRxiv*10.1101/2020.11.17.387134 (2021). 10.1186/s13059-021-02572-z PMC8734071 34991667

