# Supplementary material for: DNA methylation aging and transcriptomic studies in horses
Source: Nat Commun. 2022 Jan 10;13:40. doi: 10.1038/s41467-021-27754-y (PMC8748428; doi:10.1038/s41467-021-27754-y)
Supplement: Supplementary file 1 — Supplementary Information [file 41467_2021_27754_MOESM1_ESM.pdf]

SUPPLEMENTARY INFORMATION
for "DNA methylation aging and transcriptomic studies in horses"
by Horvath, Haghani et al.

SUPPLEMENTARY FIGURES

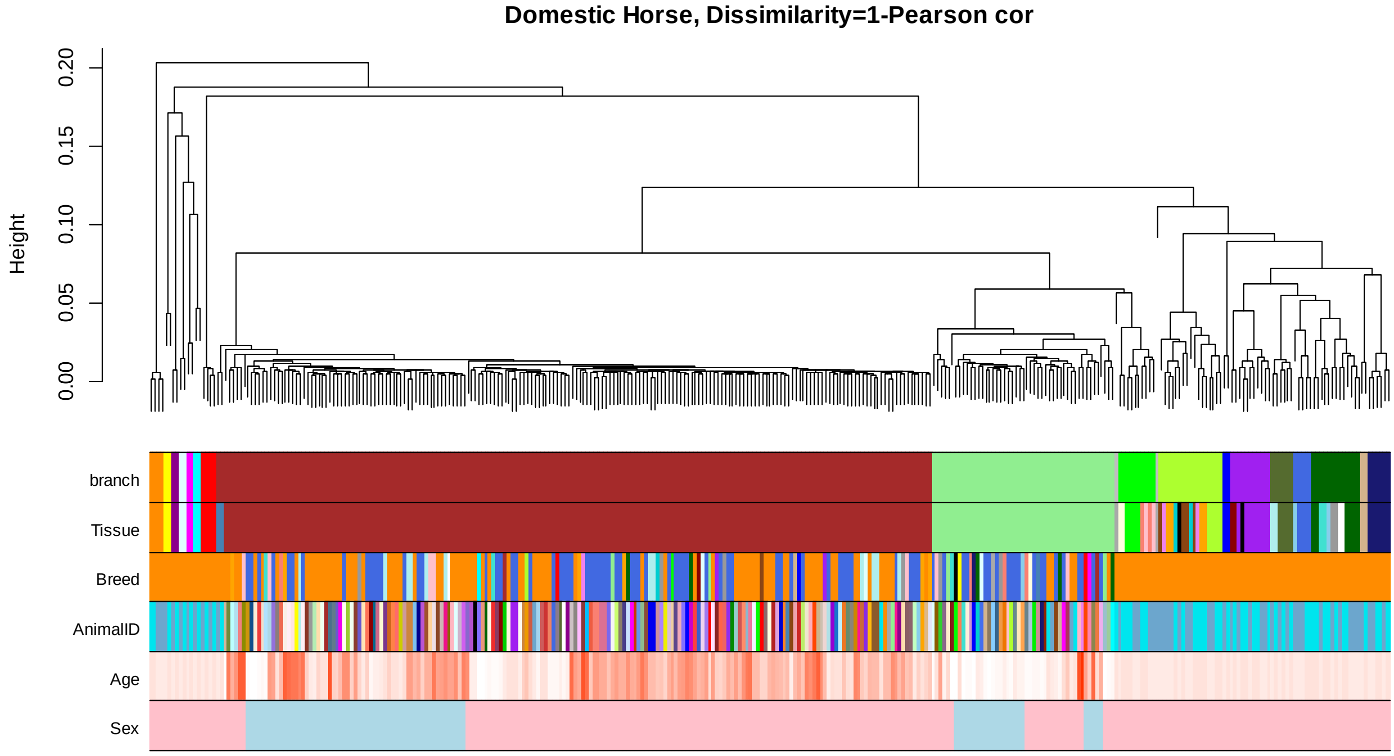

Supplementary Figure S1. Unsupervised hierarchical clustering of samples from domestic horses. Average linkage hierarchical clustering based on the inter-array correlation coefficient (Pearson correlation). The cluster branches (first color band) correspond to tissue type (second color band): brown=blood, lightgreen=liver, purple=heart, midnightblue=kidney, darkorange=pituitary gland. Third color band encodes horse breed (darkorange=thorough bred, royalblue=Quarter Horse, paleturquoise=Warmblood, pink=Hanoverian. Fourth color band visualized the animal ID. Age encodes chronological age (red=old age). Sex encodes female (pink) and male (lightblue). The figure uses N=333 arrays/tissue samples.

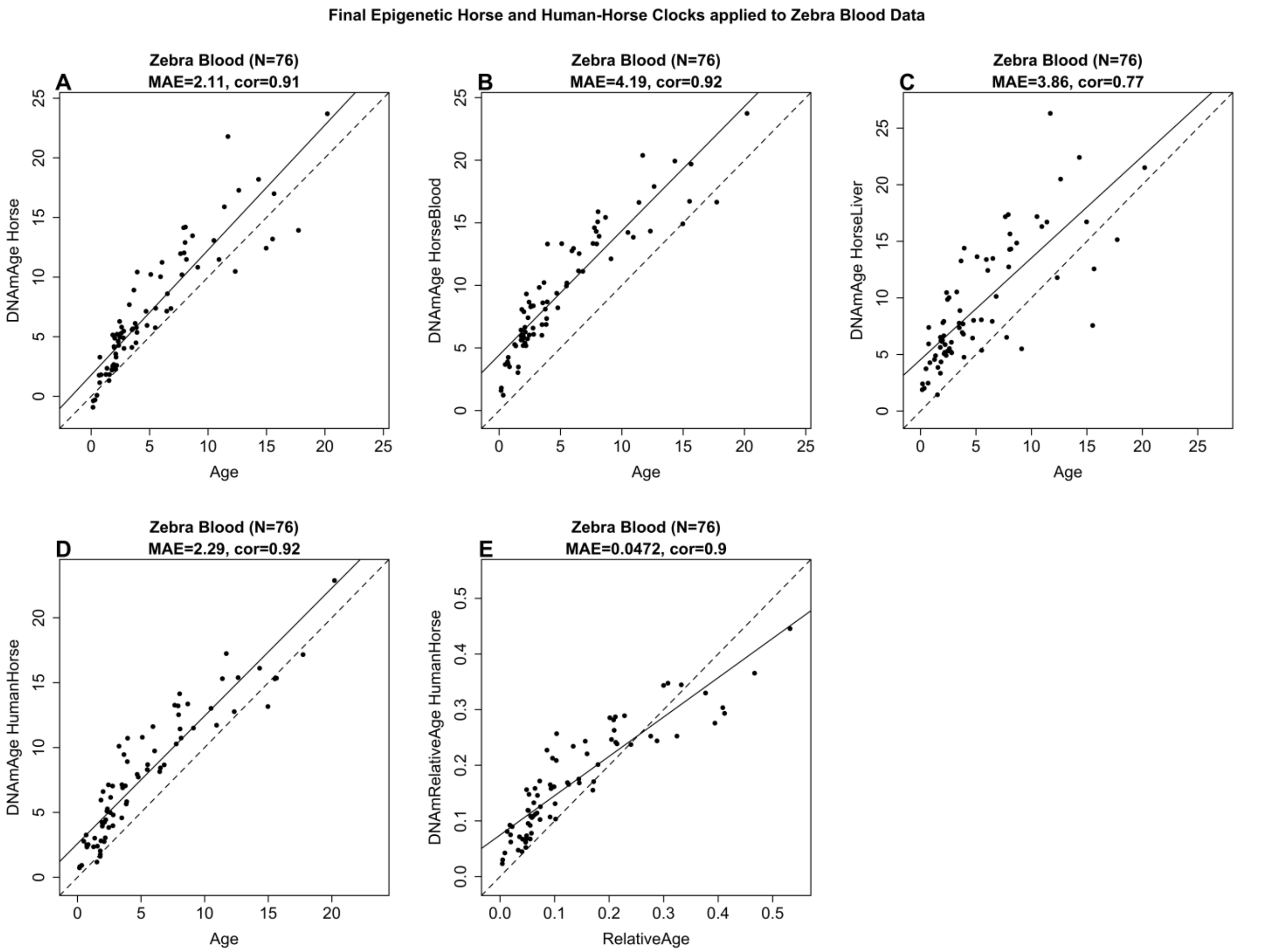

Figure S2. Horse clocks applied to blood samples from plains zebra. Each dot corresponds to a blood sample from zebras (N=76). Each panel reports the results from a different horse clock (y-axis): A) Multi-tissue clock, B) blood clock, C) liver clock, D) human-horse clock for chronological age, E) human-horse clock for relative age. DNA methylation based age estimates and chronological age are in units of years. Relative age is a number between 0 and 1. The solid line corresponds to linear least squares regression and the diagonal line to y=x. Each panel reports the number of blood samples (N=76), the median absolute error, and the Pearson correlation coefficient.

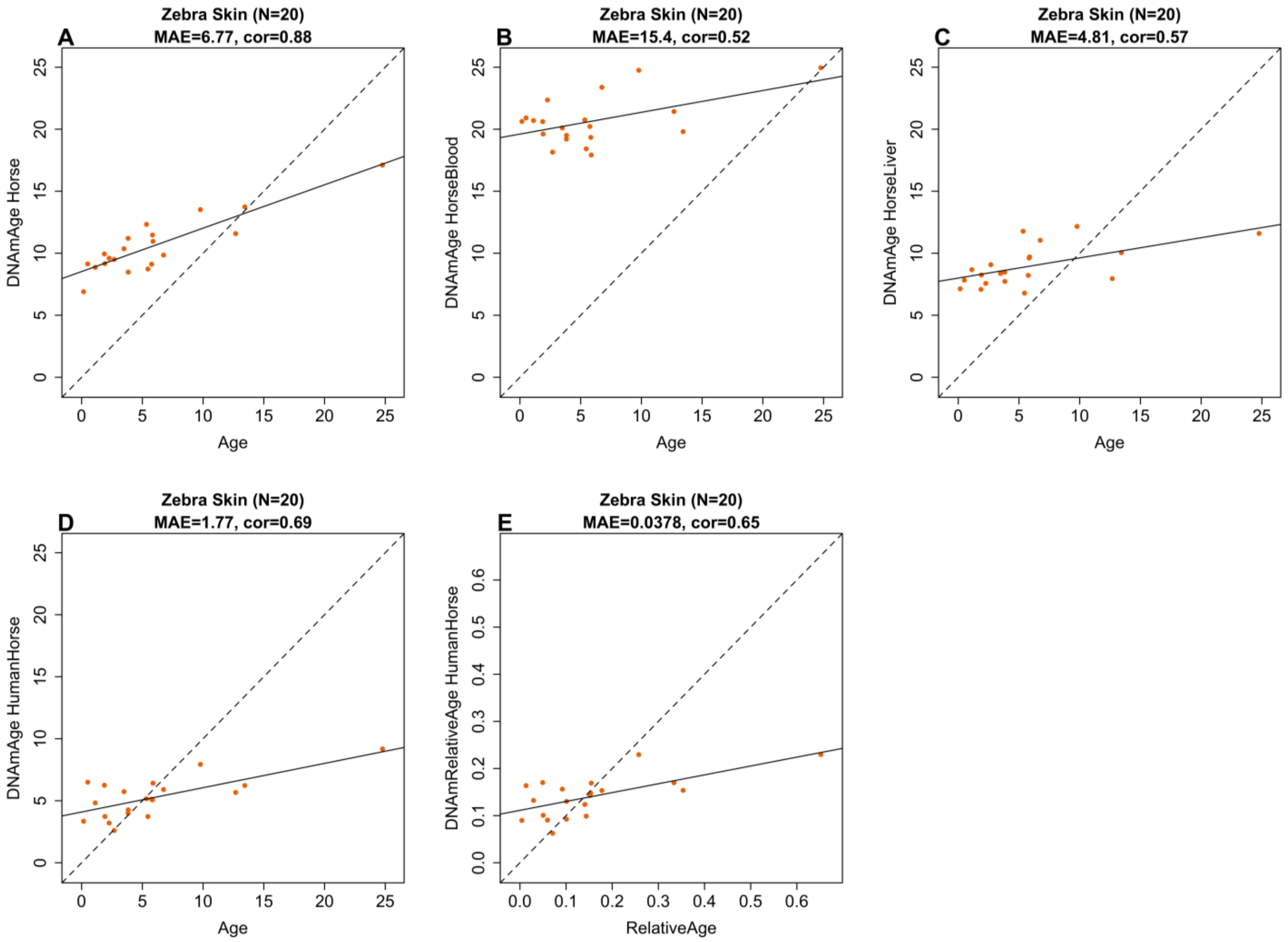

Figure S3. Horse clocks applied to skin samples from plains zebra.
Dots correspond to skin samples from zebras. Each panel corresponds to a different horse clock (y-axis): A) Multi-tissue clock, B) blood clock, C) liver clock, D) human-horse clock for chronological age, E) human-horse clock for relative age The solid line corresponds to linear least squares regression and the diagonal line to y=x. Each panel reports the number of samples (N=20), the median absolute error and the Pearson correlation coefficient.

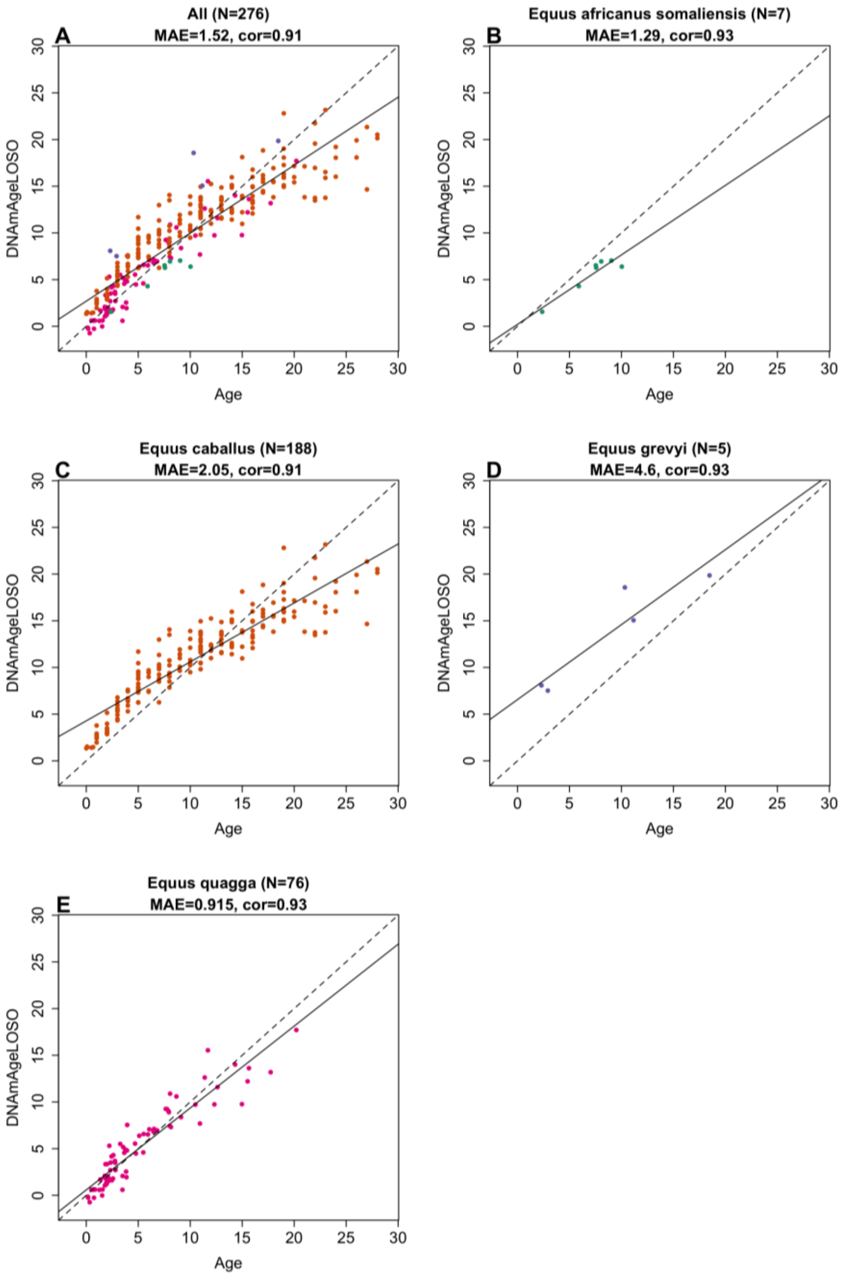

Figure S4. Leave-one-species out (LOSO) analysis of equid clock for blood samples. The LOSO cross validation method is used to evaluate to what extend the equid clock generalizes to equid species that were not part of the training set. The cross validation schemes cycles through species. For each left out species (test set) a new equid clock is trained on the remaining equid species. Next the resulting clock equid clock is evaluated in the left out equid species to arrive at an unbiased DNAm based estimate. B-E) each panel corresponds to a different equid species considered as test set. Each panel reports the number of blood samples (N), the median absolute error (in units of years) and the Pearson correlation coefficient (cor). Cross validation estimate of age (y-axis) versus chronological age in A) all species combined, B) *Equus africanus somaliensis*, C) *Equus caballus*, D) *Equus grevyi*, E) *Equus quagga*. Blood samples (dots) are colored by species as indicated in the respective panels.

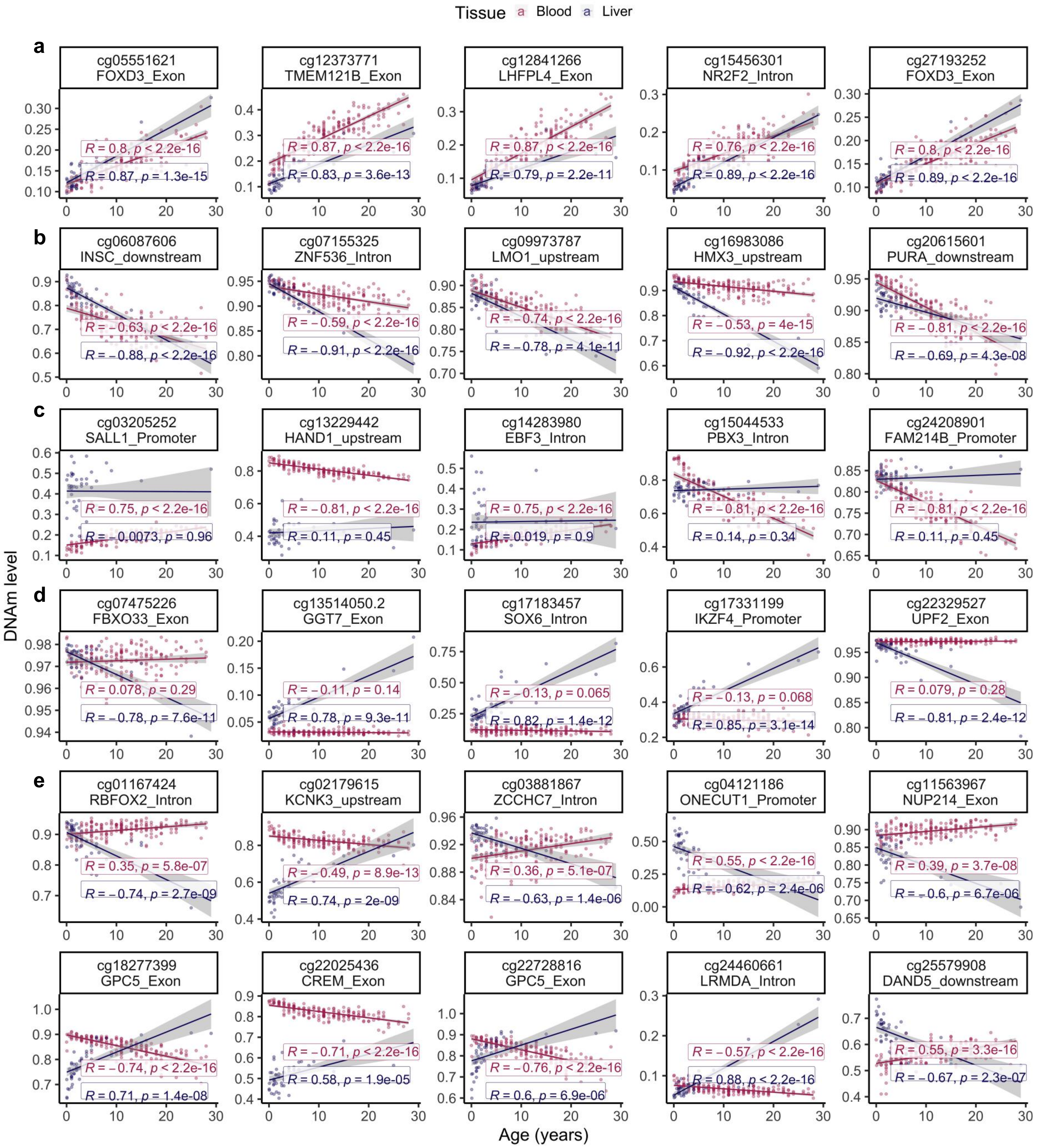

Figure S5. **Scatter plots of age-related changes in horse blood and liver**. A) CpGs that gain methylation with age in both blood and liver. B) CpGs that lose methylation with age in both blood and liver. C) Examples of blood specific changes. D) Examples of liver specific changes. E) Select CpGs with divergent aging patterns between blood and liver. Sample sizes: N=192 blood and N=48 liver samples. The panels report Pearson correlation coefficients and corresponding two sided p values (Student T test). The shading is the 95% confidence interval of the linear regression.

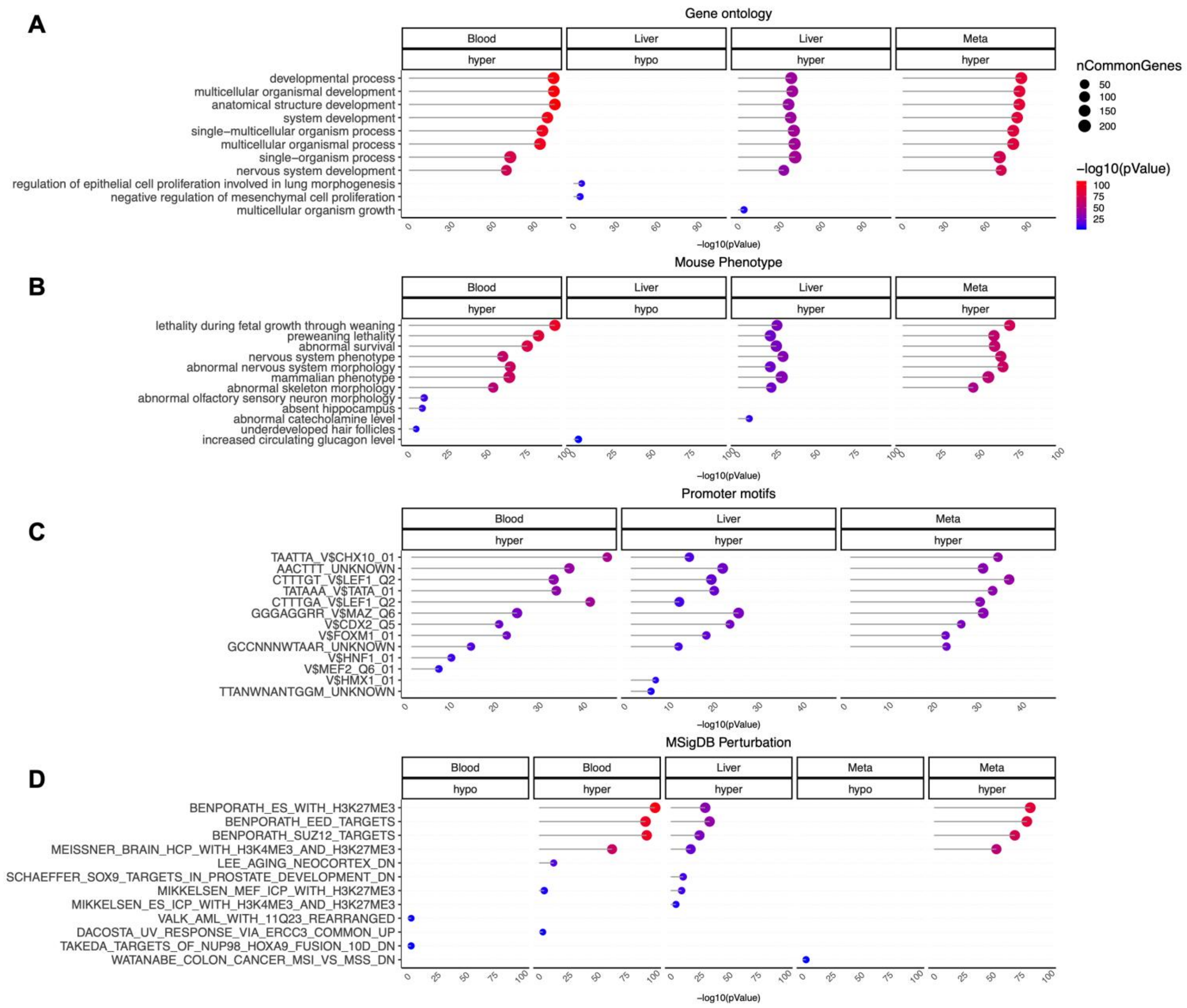

Figure S6. Gene set enrichment analysis of EWAS of age in different horse tissues. The gene level enrichment study was conducted with the GREAT software [1]. As statistical background we used all genes on the mammalian array that map to the horse genome and the human Hg19 background. Datasets: gene ontology (A), mouse phenotypes[2] (B), promoter motifs [1,3] (C), and MSigDB Perturbation [4,5] (D). The results were filtered for significance at a nominal significance level of $p < 10^{-5}$. We only report findings that led to significant enrichments, e.g. age related loss of methylation in blood (blood hypo) was omitted. The GREAT software was used to calculate one sided hypergeometric nominal (uncorrected) p values whose values are color coded as indicated in the legend.

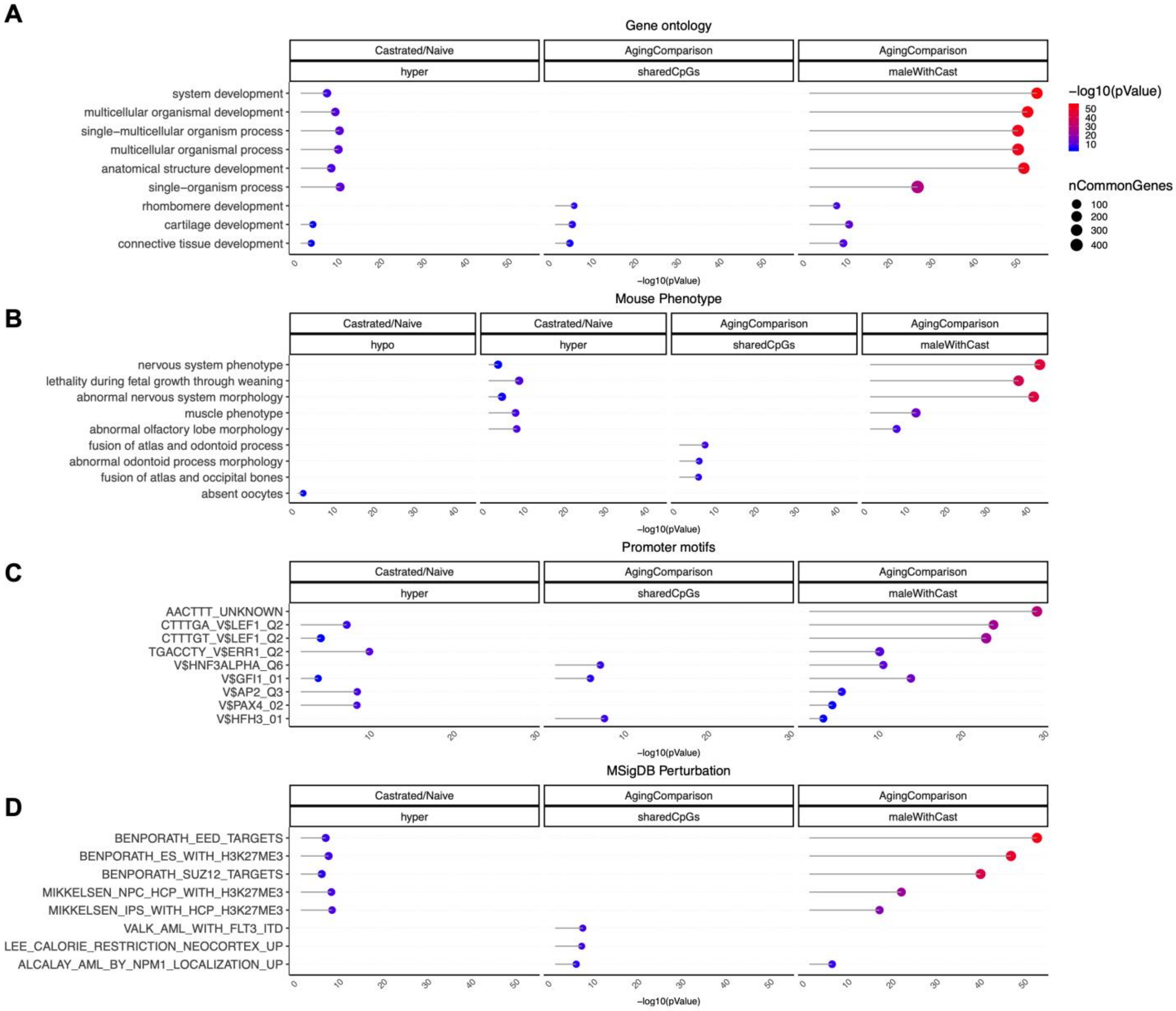

Figure S7. Gene set enrichment analysis of DNA methylation changes by castration. The gene level enrichment was done using GREAT analysis [1] and human Hg19 background. Datasets: gene ontology (A), mouse phenotypes (B), promoter motifs (C), and MSigDB Perturbation (D). The results were filtered for significance at $p < 10^{-3}$. The GREAT software was used to calculate one sided hypergeometric nominal (uncorrected) p values whose values are color coded as indicated in the legend.

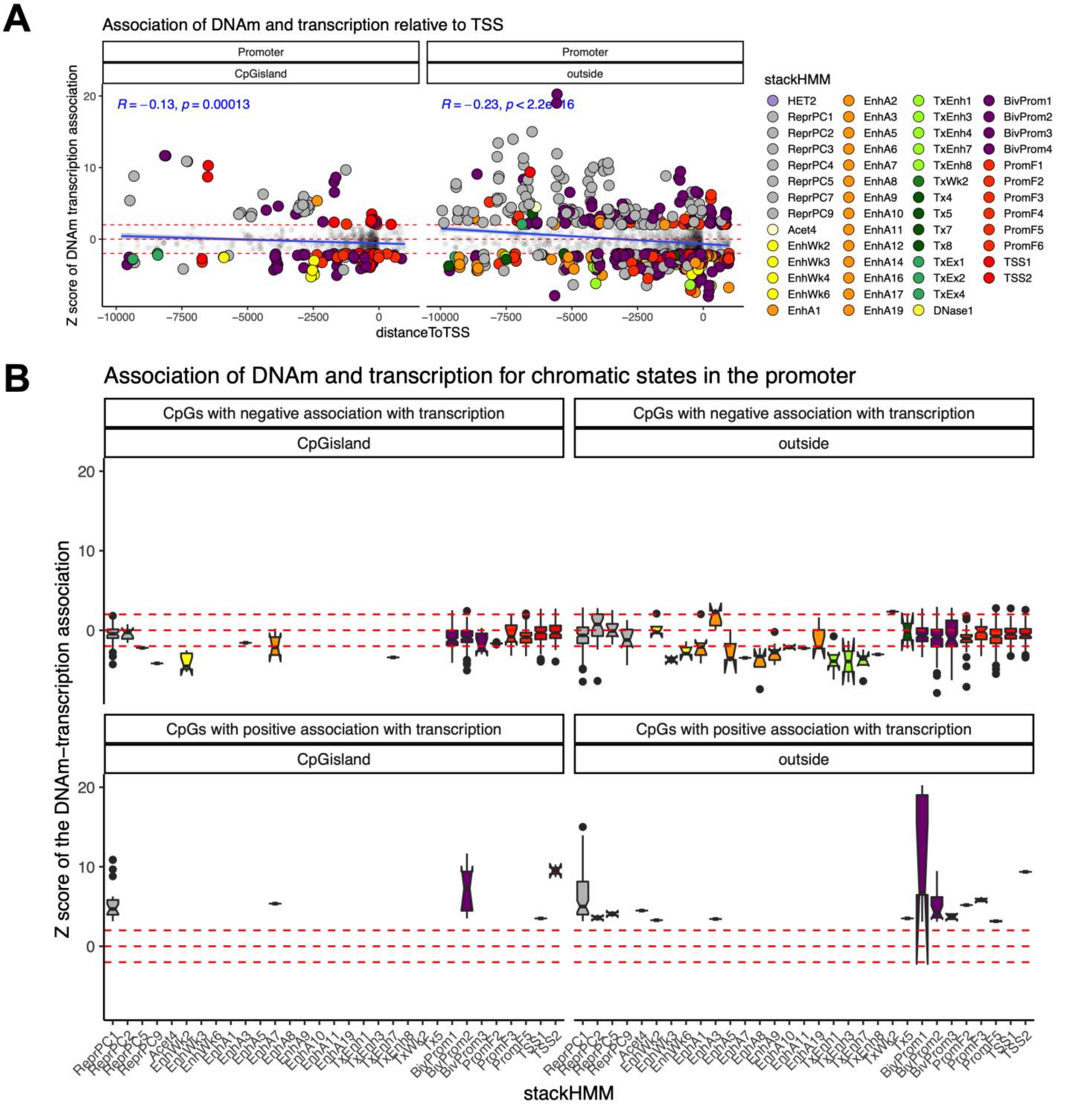

Figure S8. Promoter CpG island status does not alter DNAm-mRNA associations. A) Relationship of DNAm, mRNA expression, distance to transcription start site, and chromatin states in the gene promoters by CpG island status. The chromatin states are based on the stackHMM annotations, which represent a consensus chromatin state in over 100 human tissues [6]. Pearson correlation and corresponding Fisher transformed Z statistic (y-axis) between CpG and adjacent mRNA levels (cis-relationship) across N=29 tissue types from two horses. Red horizontal lines corespond to Z= 2 and Z= -2 (two sided significance level of 0.05). B) Boxplots of Z statistics (between CpG and mRNA) versus chromatin states (stackHMM) [7]. Each panel corresponds to different input sets of CpGs with significant cis relationship between. The input sets are distinguished by 2 criteria: 1) positive/negative association with mRNA and 2) located inside/outside of

CpG island status. Boxes show the interquartile range of the z scores. The notches indicate the 95% confidence interval of the median. The whiskers represent 1.5*IQR length of the zscores. We only present chromatin states with significant associations (p=0.05).

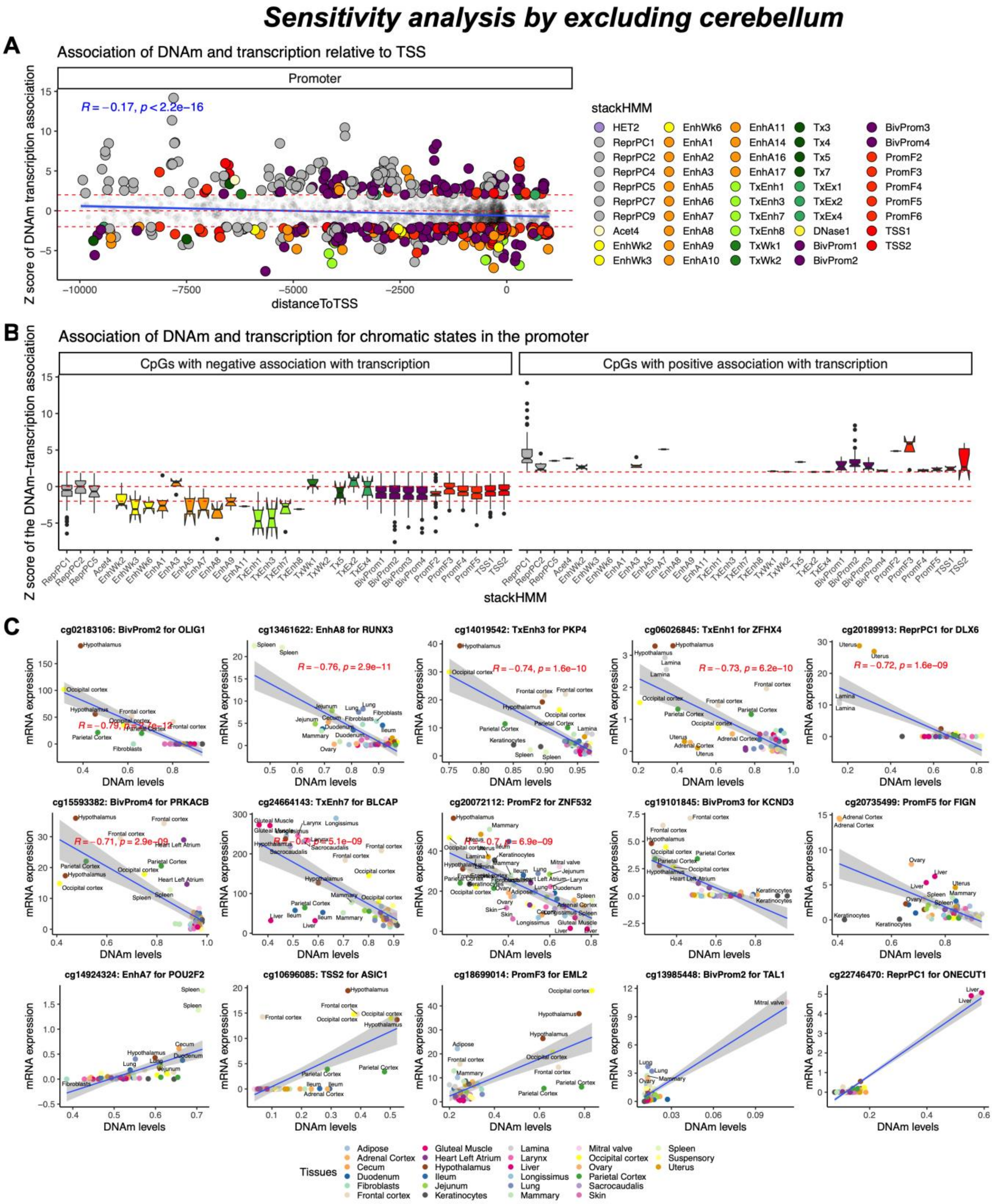

Figure S9. Sensitivity of analysis DNAm-mRNA association. Since cerebellum was a tissue with extreme DNAm-mRNA expression signatures in some stackHMM states, the cerebellum was excluded from the analysis. A) Negative association of distance to TSS with DNAm-mRNA expression association was not affected by excluding the cerebellum sample. The Z statistics are the Fisher z-transformation of DNAm-mRNA Pearson correlation for each CpG. B) Excluding the cerebellum did not affect stackHMM relationships with DNAm-mRNA changes. Boxes show the interquartile range of the z scores. The notches indicate the 95% confidence interval of the median. The whiskers represent 1.5*IQR length of the zscores. C) Scatter plots of select CpGs with DNAm-mRNA association in horse tissues after excluding cerebellum from the analysis. Pearson correlation and corresponding two sided Student T test p values for relating DNAm and mRNA level of the adjacent genes across N=27 tissue types. The shading is the 95% confidence interval of the linear regression.

Technical Details surrounding the DNAm age estimator

<u>Statistical methods used for building the clocks</u>

Each epigenetic clock was developed by fitting elastic net regression model analysis (R function glmnet) to the respective training data set. We chose the following parameters for the glmnet R function (alpha: 0.5, CV Fold: 10, Lambda choice for Clock: 1 standard error above minimum CV-MSE).

<u>Covariates and coefficient values of the horse clocks</u>

The CpGs and coefficient values can be found in **Supplementary Supplementary Data 10**.

1) The horse multi tissue clock (trained in blood and liver samples) is based on 97 CpGs whose coefficient values are specified in the column "Coef.HorseMultiTissue". Age transformation=identity, i.e. F(Age)=Age
2) The horse BLOOD tissue clock is based on 60 CpGs whose coefficient values are specified in the column "Coef.HorseBlood". Age transformation=identity, i.e. F(Age)=Age
3) The horse LIVER tissue clock is based on 42 CpGs whose coefficient values are specified in the column "Coef.HorseBlood". Age transformation=identity, i.e. F(Age)=Age
4) The human horse clock for chronological age is based on 435 CpGs whose coefficient values are specified in the column "Coef.HumanHorseLogLinearAge". Age transformation=log-linear described below.
5) The human horse clock for relative age is based on 510 CpGs whose coefficient values are specified in the column "Coef.HumanHorseRelativeAge". Age transformation: relative age. i.e. F(Age)=Age/maxLifespan. Max lifespan for horses is 57 years. Human max lifespan =122.5 years.
6) The epigenetic clocks for blood samples from equids (column Coef.EquidBloodLogLinearAge) uses 63 CpGs. Age transformation=log-linear described below.

General description of age transformation

The human-horse clocks for chronological age used log linear transformations that are similar to those employed for the HUMAN pan tissue (Horvath 2013) [8].

An elastic net regression model (implemented in the glmnet R function) was used to regress a transformed version of age on the beta values in the training data. The glmnet function requires the user to specify two parameters (alpha and beta). Since I used an elastic net predictor, alpha was set to 0.5. But the lambda value of was chosen by applying a 10 fold cross validation to the training data (via the R function cv.glmnet).

The elastic net regression results in a linear regression model whose coefficients b_0, b_1, . . . , relate to transformed age as follows

F(chronological age)=b_0+$b_1 CpG_1$+ . . . +$b_p CpG_p$+error

Note that the intercept term is denoted by b_0. The coefficient values can be found in the attached Excel file.

Based, on the coefficient values from the regression model, DNAmAge is estimated as follows

*DNAm*Age=F^{-1}(b_0+$b_1 CpG_1$+ . . . +$b_p CpG_p$)

where $F^{-1}(y)$ denotes the mathematical inverse of the function F(.). Thus, the regression model can be used to predict to transformed age value by simply plugging the beta values of the selected CpGs into the formula.

Defining Properties of the log linear transformation

As indicated by its name, the "log-linear" function, has a logarithmic dependence on age before the average age of sexual maturity (of the species) and a linear dependence after Age at Sexual Maturity (of the species). For the human-horse clocks we used the following averages at sexual maturity (in units of years): 13.5 years for humans and 2.58493 years for horses.

Construction

We used a piecewise transformation, parameterized by Age of Sexual Maturity (A).

The transformation is F(x), given by

$$F(x) = g\left(\frac{x+1.5}{A+1.5}\right) \text{ where } g(t) = \begin{cases} \log(t), & for\ 0 \le t \le 1 \\ t-1, & for\ 1 \le t \end{cases}$$

Explicitly, F(x) is given by

$$F(x) = \begin{cases} \log\left(\frac{x+1.5}{A+1.5}\right), & for\ 0 \le x \le A \\ \frac{x-A}{A+1.5}, & for\ A \le x \end{cases}$$

In order to use this transformation to predict Age on <u>*new samples*</u>, one needs to use the *inverse* transformation, $F^{-1}(y)$, given by

$$F^{-1}(y) = \begin{cases} (A+1.5)*\exp(y) - 1.5, & for\ y \le 0 \\ (A+1.5)y + A, & for\ y \ge 0 \end{cases}$$

For predicting age, apply the inverse transformation to coefficient-weighted sum. That is,

$$DNAmAge = F^{-1}(x * \beta)$$

where β is the vector of coefficients and x is the vector of methylation values, with an intercept term.

The DNAm Age estimate is estimated in two steps.

First, one forms a weighted linear combination of the CpGs whose details can be found in Table
 The table reports the probe identifier (cg number) used in the custom Infinium array (HorvathMammalMethylChip40) . The weights used in this linear combination are specified in the respective column entitled "Coef.".

The formula assumes that the DNA methylation data measure "beta" values but the formula could be adapted to other ways of generating DNA methylation data.

<u>Species characteristics of Equids according to anAge</u>

For the sake of reader friendliness, we reproduce select species characteristics from the anAge data base about different equid species. Our age transformations make use of column "AgeSexualMaturity.Years", which is the average across male and female age at sexual maturity. The variable names indicate the units of time, e.g Days or Years.

The relative age estimate makes use of the maxLifespan which is in units of years.

Species	Latin Name	GestationTime.days	maxLifespan.Years	AgeSexualMaturity.Years	Female.maturity.days	Male.maturity.days	AverageAdultWeight
African wild ass	Equus asinus	359	47	2.347	708	1005	1.65E+05
Horse	Equus caballus	337	57	2.585	914	973	3.00E+05
Grevy's zebra	Equus grevyi	406	31	3.752	1278	1461	3.84E+05
Kulan	Equus hemionus	339	31.6	3.211	1157	1187	2.30E+05
Kiang	Equus kiang	299	30.1	NA	NA	NA	2.75E+05
Quagga	Equus quagga	365	38	2.466	900	900	2.80E+05
Mountain zebra	Equus zebra	362	33.2	3.134	1009	1279	2.96E+05

Supplementary Table 1. Characteristics of equid species. Rows correspond to different equid species in this article. Columns report gestation time (in units of days), maximum lifespan (in units of years), age at sexual maturity averaged across both sexes (in units of years), female age at sexual maturity (in units of days), male age at sexual maturity (in units of days), average adult weight (in grams). These values come from the anAge data base.

The DNAm Age estimate is estimated in two steps.

First, one forms a weighted linear combination of the CpGs whose details can be found in the supplementary Excel file (**Supplementary Data 10**)
The file reports the probe identifier (cg number) used in the custom Infinium array (HorvathMammalMethylChip40). The weights used in this linear combination are specified in the respective column entitled "Coef.".
The formula assumes that the DNA methylation data measure "beta" values but the formula could be adapted to other ways of generating DNA methylation data.

Supplementary Note 1.

R software code for horse clocks

R Implementation of the log linear transformation

```
### Applies the log linear transformation to the input vector x,i.e. to Age
F= Vectorize(function(x, maturity, ...) {
  if (is.na(x) | is.na(maturity)) {return(NA)}
  k <- 1.5
  y <- 0
  if (x < maturity) {y = log((x+k)/(maturity+k))}
  else {y = (x-maturity)/(maturity+k)}
  return(y)
})
### Inverse log linear trnasformation
F.inverse= Vectorize(function(y, maturity, ...) {
  if (is.na(y) | is.na(maturity)) {return(NA)}

  k <- 1.5
  x <- 0
  if (y < 0) {x = (maturity+k)*exp(y)-k}
  else {x = (maturity+k)*y+maturity}
  return(x)
})

# R function for multivariate regression model
multivariatePredictorCoef=function(dat0, datCOEF,imputeValues=FALSE) {
datout=data.frame(matrix(NA,nrow=dim(dat0)[[2]]-1,ncol=dim(datCOEF)[[2]]-1 ))
match1=match(datCOEF[-1,1],dat0[,1] )
if (   sum(!is.na(match1))==0 ) stop("Input error. The first column of dat0 does not contain CpG identifiers (cg numbers).")
dat1=dat0[match1,]
row.names1=as.character(dat1[,1])
dat1=dat1[,-1]
if (imputeValues ){dat1=impute.knn(data=as.matrix(dat1) ,k = 10)[[1]]}
for (i in 1:dim(dat1)[[2]] ){ for (j in 2:dim(as.matrix(datCOEF))[[2]] ){
datout[i,j-1]=sum(dat1[,i]* datCOEF[-1,j],na.rm=TRUE)+ datCOEF[1,j]}}
colnames(datout)=colnames(datCOEF)[-1]
rownames(datout)=colnames(dat0)[-1]
datout=data.frame(SampleID= colnames(dat0)[-1],datout)
datout
} # end of function

# read in supplementary table 10
datCoef=read.csv("SupplementaryData10.csv")
```

The first columns should read as follows

```
names(datCoef)
```

```
[1] "var"
[2] "Coef.HorseMultiTissue"
[3] "Coef.HorseBlood"
[4] "Coef.HorseLiver"
[5] "Coef.HumanHorseAgeLogLinear"
[6] "Coef.HumanHorseRelativeAge"
[7] "Coef.EquidBloodAgeLogLinear"
```

```
# Restrict attention to the first 7 columns
datCoef=datCoef[,c(1:7)]

match1=match(datCoef[-1,1],dat0[,1] )
missingProbes= as.character(datCoef[-1,1] )[is.na(match1)]

dat1=dat0[match1,]
# data frame with predicted values.
datPredictions=multivariatePredictorCoef(dat1,datCOEF=datCoef,imputeValues=FALSE)

#let's relabel the columns by replacing "Coef" with "DNAm" since the columns contain estimates of age or relative age instead of coefficient values

colnames(datPredictions)=gsub(pattern="Coef", replacement="DNAm", x=colnames(datPredictions))
# We need to transform the human horse clock for chronological age using the inverse of the log linear transformation.
```

For data from horses, the age at sexual maturity has to be set to 2.585 years.

```
datPredictions$DNAm.HumanHorseAgeLogLinear=
F.inverse(datPredictions$DNAm.HumanHorseAgeLogLinear, maturity= 2.585)
datPredictions$DNAm.EquidBloodAgeLogLinear=
F.inverse(datPredictions$DNAm.EquidBloodAgeLogLinear, maturity= 2.585)
```

The data frame "datPredictions" contains the age estimates in units of years and relative age estimates.

References

1 McLean, C. Y. *et al.* GREAT improves functional interpretation of cis-regulatory regions. *Nat Biotechnol* **28**, doi:10.1038/nbt.1630 (2010).

2 Eppig, J. T., Blake, J. A., Bult, C. J., Kadin, J. A. & Richardson, J. E. The Mouse Genome Database (MGD): facilitating mouse as a model for human biology and disease. *Nucleic Acids Res* **43**, D726-736, doi:10.1093/nar/gku967 (2015).

3 Yu, G., Wang, L.-G. & He, Q.-Y. ChIPseeker: an R/Bioconductor package for ChIP peak annotation, comparison and visualization. *Bioinformatics* **31**, 2382-2383, doi:10.1093/bioinformatics/btv145 (2015).

4 Subramanian, A. *et al.* Gene set enrichment analysis: A knowledge-based approach for interpreting genome-wide expression profiles. *Proceedings of the National Academy of Sciences* **102**, 15545-15550, doi:10.1073/pnas.0506580102 (2005).

5 Liberzon, A. *et al.* Molecular signatures database (MSigDB) 3.0. *Bioinformatics* **27**, 1739-1740, doi:10.1093/bioinformatics/btr260 (2011).
6 Vu, H. & Ernst, J. Universal annotation of the human genome through integration of over a thousand epigenomic datasets. *bioRxiv*, 2020.2011.2017.387134, doi:10.1101/2020.11.17.387134 (2020).
7 Vu, H. & Ernst, J. Universal annotation of the human genome through integration of over a thousand epigenomic datasets. *bioRxiv*, 2020.2011.2017.387134, doi:10.1101/2020.11.17.387134 (2021).
8 Horvath, S. DNA methylation age of human tissues and cell types. *Genome Biol* **14**, R115, doi:10.1186/gb-2013-14-10-r115 (2013).
