# Supplementary material for: DNA methylation aging and transcriptomic studies in horses
Source: Nat Commun. 2022 Jan 10;13:40. doi: 10.1038/s41467-021-27754-y (PMC8748428; doi:10.1038/s41467-021-27754-y)
Supplement: Supplementary file 2 — Description of Additional Supplementary Files [file 41467_2021_27754_MOESM2_ESM.pdf]

Description of Additional Supplementary Files

for "DNA methylation aging and transcriptomic studies in horses"

by Horvath, Haghani et al.

File Name: Supplementary Data 1
Description: Top age related CpGs in horse blood, horse liver, and Stouffer meta-analysis across tissues. The EWAS was done by Pearson correlation and two-sided Student t-test. The significant top hits are selected for up to 1000 CpGs (500 per direction of association) at $p<10^{-5}$ significance. Related to Figure 3.

File Name: Supplementary Data 2
Description: GREAT enrichment results of EWAS of age in different horse tissues. The gene level enrichment was done using human Hg19 background with 50 kb window for annotating the loci. The results are limited to terms in all datasets with hypergeometric p-value<0.005. Related to Supplementary Figure S6.

File Name: Supplementary Data 3
Description: EWAS of age and Transcriptomic Wide Association Study of age (TWAS) in horse peripheral blood mononuclear cells. Age related mRNA changes in horse is based on PBMC samples from a publicly available dataset (GSE101117). The EWAS was done by Pearson correlation and two-sided Student t-test in horse blood. The EWAS results are significant at a two-sided $p<10^{-5}$. Related to Figure 3.

File Name: Supplementary Data 4
Description: EWAS of age in the blood of castrated and intact stallions. The EWAS was done by Pearson correlation and two-sided Student t-test. The significant top hits are selected for up to 1000 CpGs (500 per direction of association) at $p<10^{-4}$ significance. Related to Figure 4.

File Name: Supplementary Data 5
Description: Top CpGs related to castration status of stallions blood. The EWAS was done by a multivariate linear regression model, including age as a co-variate. The significant top hits are selected for up to 1000 CpGs (500 per direction of association) at $p<10^{-4}$ significance. Related to Figure 4.

File Name: Supplementary Data 6
Description: Great enrichment analysis of CpGs that relate to castration status. The gene level enrichment was done using human Hg19 background with 50 kb window for annotating the loci. The results are limited to terms in all datasets with hypergeometric p-value<0.005. Related to Supplementary Figure S7.

File Name: Supplementary Data 7
Description: Explanation of Chromatin States (Stack Hidden Markov Model). These “stacked chromatin states (stackHMM)” identifies chromatin features based on the

consensus of over 100 human cell types. Related to Figure 5 and Supplementary Figure S8.

File Name: Supplementary Data 8
Description: Correlation between CpGs and mRNA from adjacent genes across horse tissues. Cis relationships. This analysis was based on a Pearson correlation of DNAm and mRNA level of the adjacent genes in 29 different tissues from two female horses. Each CpG was assigned to one gene based on the closest distance to the transcriptional start sites. Related to Figure 5 and Supplementary Figure S8.

File Name: Supplementary Data 9
Description: Description of human tissues used for building the human-horse clocks. Related to Figure 1.

File Name: Supplementary Data 10
Description: Coefficient values and CpGs of the different epigenetic clocks for horses and equids. Related to Figure 1 and Figure 2.
