# Supplementary material for: DNA methylation aging and transcriptomic studies in horses
Source: Nat Commun. 2022 Jan 10;13:40. doi: 10.1038/s41467-021-27754-y (PMC8748428; doi:10.1038/s41467-021-27754-y)
Supplement: Supplementary file 4 — Reporting Summary [file 41467_2021_27754_MOESM4_ESM.pdf]

nature research

Corresponding author(s): Steve Horvath, Carrie Finno

Last updated by author(s): November 25, 2021

Reporting Summary

Nature Research wishes to improve the reproducibility of the work that we publish. This form provides structure for consistency and transparency in reporting. For further information on Nature Research policies, see our Editorial Policies and the Editorial Policy Checklist.

Statistics

For all statistical analyses, confirm that the following items are present in the figure legend, table legend, main text, or Methods section.

n/a	Confirmed	
☐	☒	The exact sample size (*n*) for each experimental group/condition, given as a discrete number and unit of measurement
☐	☒	A statement on whether measurements were taken from distinct samples or whether the same sample was measured repeatedly
☐	☒	The statistical test(s) used AND whether they are one- or two-sided<br>*Only common tests should be described solely by name; describe more complex techniques in the Methods section.*
☐	☒	A description of all covariates tested
☐	☒	A description of any assumptions or corrections, such as tests of normality and adjustment for multiple comparisons
☐	☒	A full description of the statistical parameters including central tendency (e.g. means) or other basic estimates (e.g. regression coefficient) AND variation (e.g. standard deviation) or associated estimates of uncertainty (e.g. confidence intervals)
☐	☒	For null hypothesis testing, the test statistic (e.g. *F*, *t*, *r*) with confidence intervals, effect sizes, degrees of freedom and *P* value noted<br>*Give P values as exact values whenever suitable.*
☒	☐	For Bayesian analysis, information on the choice of priors and Markov chain Monte Carlo settings
☒	☐	For hierarchical and complex designs, identification of the appropriate level for tests and full reporting of outcomes
☐	☒	Estimates of effect sizes (e.g. Cohen's *d*, Pearson's *r*), indicating how they were calculated

Our web collection on statistics for biologists contains articles on many of the points above.

Software and code

Policy information about availability of computer code

Data collection	The data base was maintained as Microsoft Excel spreadsheet (Excel 2013).
Data analysis	Microsoft Excel 2013 for data management and collection.<br>GREAT software version 4.0.4<br>R_4.0.2: Programming language for statistical computing<br>R_Chipseeker_1.8.6: Determine distance of CpG to nearest transcription start site<br>R_QuasR_1.30.0: Align probe sequences to genome assembly<br>R_sesame_1.3.0: Normalize Illumina Infinium DNA methylation array data<br>ART_2.0: Predicts functional factors that bind at cis-regulatory regions to regulate gene expression<br>Bedtools_2.25.0: Evaluate overlap between probe sets and transcription factor motifs on array<br>JMP Pro_14.1: Predictive analytics software for statistical analysis and visualization<br>METAL_03-25-11: Facilitates meta-analysis of large datasets in a memory efficient manner<br>PANTHER_16: Gene Ontology enrichment analysis<br>R_4.0.2: Programming language for statistical computing<br>R_glmnet_4.0-2: Fits lasso or elastic-net generalized linear models<br>R_phyper_3.5.1: Probability for the hypergeometric distribution<br>R_samtools_2.6.0: Utilities for manipulating sequence alignments<br>R_WGCNA_1.69: Weighted correlation network analysis

For manuscripts utilizing custom algorithms or software that are central to the research but not yet described in published literature, software must be made available to editors and reviewers. We strongly encourage code deposition in a community repository (e.g. GitHub). See the Nature Research guidelines for submitting code & software for further information.

Data

Policy information about availability of data

All manuscripts must include a data availability statement. This statement should provide the following information, where applicable:

- Accession codes, unique identifiers, or web links for publicly available datasets
- A list of figures that have associated raw data
- A description of any restrictions on data availability

The methylation data from horses, zebras, and equids generated in this study have been deposited in Gene Expression Omnibus (accession numbers GSE174767, GSE184222, GSE184223). The RNA-seq data can be downloaded from https://www.ebi.ac.uk/ena/data/view/ERA1487553
The human methylation data were not generated for this study. These data will be presented in another publication and can be requested from SH. In addition, the data will be posted on GEO as part of the data release from the Mammalian Methylation Array Consortium.
The mammalian methylation array is available through the non-profit Epigenetic Clock Development Foundation (https://clockfoundation.org/).
We used species characteristics from the AnAge Data base https://genomics.senescence.info/species/

Field-specific reporting

Please select the one below that is the best fit for your research. If you are not sure, read the appropriate sections before making your selection.

☒ Life sciences ☐ Behavioural & social sciences ☐ Ecological, evolutionary & environmental sciences

For a reference copy of the document with all sections, see nature.com/documents/nr-reporting-summary-flat.pdf

Life sciences study design

All studies must disclose on these points even when the disclosure is negative.

Sample size

We aimed to develop epigenetic clocks for blood samples from horses and other equids. A second goal was to study how methylation levels correlate with transcriptional data across different tissue types from horses. To address these goals we generated DNA methylation data from all tissue samples that were available in our freezers. We generated DNA methylation data from n=333 horse tissue samples. Blood samples were collected via venipuncture into EDTA tubes from across 24 different horse breeds (buffy coat). The other tissues were collected at necropsy. The tissue atlas was generated from two Thoroughbred mares as part of the FAANG initiative 37, with the following tissues profiled: adipose (gluteal), adrenal cortex, blood (PBMCs; only n=1 mare), cartilage (only n=1 mare), cecum, cerebellum (2 samples each from lateral hemisphere and vermis), frontal cortex, duodenum, fibroblast, heart (2 samples each from the right atrium, left atrium, right ventricle, left ventricle), hypothalamus, ileum, jejunum, keratinocyte, kidney (kidney cortex and medulla), lamina, larynx (i.e. cricoarytenoideus dorsalis muscle), liver, lung, mammary gland, mitral valve of the heart, skeletal muscle (gluteal muscle and longissimus muscle), occipital cortex, ovary, parietal cortex, pituitary, sacrocaudalis dorsalis muscle, skin, spinal cord (C1 and T8), spleen, suspensory ligament, temporal cortex, tendon (deep digital flexor tendon and superficial digital flexor tendon), uterus.

Additional equid species
The data from the 3 additional equid species are also described in a companion paper (B. Larison 2021, Communications in Biology) that focuses on plains zebras (Equus quagga). Briefly, both blood (n=96) and biopsy (skin) (n=24) samples from plains zebras were obtained from a captive population of zebras maintained in a semi-wild state by the Quagga Project (Harley, 2009) in the Western Cape of South Africa. The population was founded in 1989 from 19 individuals (9 from Etosha National Park in Namibia, 10 from the Kwazulu-Natal in South Africa). Skin samples were taken by remote biopsy dart (1 mm wide by 20-25 mm deep plug) and preserved in RNAlater (Qiagen). Blood samples were taken opportunistically during veterinarian visits and preserved in EDTA tubes. Most samples were collected from different individuals, except for two animals that were sampled twice some years apart. All samples were stored at -20 °C. Samples were collected under a protocol approved by the Research Safety and Animal Welfare Administration, University of California Los Angeles: ARC # 2009-090-31, originally approved in 2009. After eliminating samples with low confidence for individual identity and age, we retained 76 blood samples, and 20 skin samples. We retained the founder, however, in an effort to extend the age range represented in the skin clock. The mean age of blood was 5.2 years ranging from 0.16 years to 20 years. The mean age of skin was 5.9 years ranging from 0.16 years to 25 years.
The Grevy's zebra (n=5) and Somali wild ass (n=7), are samples from zoo-based animals that were opportunistically collected and banked during routine health exams and the DNA methylation profiles from these samples have been reported previously (Larison 2021).

Sample size calculations. The N=192 blood samples from horses provid 80% power to detect a correlation as low as r=0.20 at a two sided significance level of 0.05 using a Pearson correlation test (Student T test). Thus, our study was well powered to detect aging effects in blood. No sample size calculation was performed for the additional equid species. Rather, we profiled all available samples from the other equids.

Data exclusions

None

Replication

The term replication is not quite appropriate since we did not carry out experiments. However, we provide several unbiased assessments of the accuracy of our age estimators in this is an observational studies. We carried out 3 cross-validation scheme for arriving at unbiased estimates of the accuracy of the different DNA methylation-based age estimators (clocks): 1) Leave-one sample-out cross validation (LOOCV) for single species clocks (e.g. horse clock). 2) Species balanced 10 fold cross validation for human-horse clocks and for equid clocks. 3) Leave-one-species out (LOSO) analysis for the equid clock for blood samples (Figures S4) in order to estimate the accuracy for species that were not part of the training set. Indirect validation of the horse epigenetic clock is afforded by applying it to zebras.

Randomization	This is an observational study, so randomization is not relevant. However, we randomize samples with respect to age, sex when designing the 96-well plates for methylation profiling.
Blinding	Blinding was not relevant to our study, because this is an observational study and all available data were used.

Reporting for specific materials, systems and methods

We require information from authors about some types of materials, experimental systems and methods used in many studies. Here, indicate whether each material, system or method listed is relevant to your study. If you are not sure if a list item applies to your research, read the appropriate section before selecting a response.

Materials & experimental systems

n/a	Involved in the study
☒	☐ Antibodies
☒	☐ Eukaryotic cell lines
☒	☐ Palaeontology and archaeology
☐	☒ Animals and other organisms
☒	☐ Human research participants
☒	☐ Clinical data
☒	☐ Dual use research of concern

Methods

n/a	Involved in the study
☒	☐ ChIP-seq
☒	☐ Flow cytometry
☒	☐ MRI-based neuroimaging

Animals and other organisms

Policy information about studies involving animals; ARRIVE guidelines recommended for reporting animal research

Laboratory animals	Horses. Most of the samples were from the Thoroughbred (TB) (n=79) and American Quarter Horse breeds (QH, n=62). For the following breeds, we had between one and six blood samples: Andalusian, Appaloosa, Arabian, Dutch Warmblood, Hanoverian, Holsteiner, Irish Sport Horse, Lipizzaner, Lusitano, mixed breed, Oldenburg, Paint or Paint cross, Percheron, Shire, Standardbred, Warmblood and Welsh Pony. The n=49 liver samples originated from necropsy collections of horses across 19 different breeds, with most of the liver samples from QHs (n=20). All collection protocols were approved by the UC Davis Institutional Animal Care and Use Committee (Protocols #20751 and 21455, respectively).<br>Human tissue and organ samples were derived from the National NeuroAIDS Tissue Consortium. Blood samples came from the Cape Town Adolescent Antiretroviral Cohort study and PEG study. Skin and other primary cells were provided by Dr. Kenneth Raj.
Wild animals	The data from the 3 additional equid species are described in a companion paper (Larison, 2021) that focuses on plains zebras (Equus quagga). Briefly, both blood (n=96) and biopsy (skin) (n=24) samples from plains zebras were obtained from a captive population of zebras maintained in a semi-wild state by the Quagga Project (Harley, 2009)n the Western Cape of South Africa. The population was founded in 1989 from 19 individuals (9 from Etosha National Park in Namibia, 10 from the Kwazulu-Natal in South Africa). Skin samples were taken by remote biopsy dart (1 mm wide by 20-25 mm deep plug) and preserved in RNAlater (Qiagen). Blood samples were taken opportunistically during veterinarian visits and preserved in EDTA tubes. Most samples were collected from different individuals, except for two animals that were sampled twice some years apart. All samples were stored at -20 °C. Samples were collected under a protocol approved by the Research Safety and Animal Welfare Administration, University of California Los Angeles: ARC # 2009-090-31, originally approved in 2009. After eliminating samples with low confidence for individual identity and age, we retained 76 blood samples, and 20 skin samples. We retained the founder, however, in an effort to extend the age range represented in the skin clock. The mean age of blood was 5.2 years ranging from 0.16 years to 20 years. The mean age of skin was 5.9 years ranging from 0.16 years to 25 years.<br>The Grevy's zebra (n=5) and Somali wild ass (n=7), are samples from zoo-based animals that were opportunistically collected and banked during routine health exams and the DNA methylation profiles from these samples have been reported previously (Larison, 2021).
Field-collected samples	Same as wild animals.
Ethics oversight	Horse: UC Davis Institutional Animal Care and Use Committee (Protocols #20751 and 21455, respectively).<br>Other equids. Research Safety and Animal Welfare Administration, University of California Los Angeles: ARC # 2009-090-31<br>Ethics approval for human tissue (RB#18-000315).

Note that full information on the approval of the study protocol must also be provided in the manuscript.
